# Selective multimodal representation and visitor appraisal in educational heritage: evidence from Chinese academies

**DOI:** 10.3389/fpsyg.2026.1926279

**Published:** 2026-10-27

**Authors:** Wenlei Niu, Qiongxuan Zeng, Feng Liang, Yingpeng Yu

**Affiliations:** 1 School of Culture and Tourism, Wuxi University of Technology, Wuxi, Jiangsu, China; 2 School of Commerce and Tourism, Chenzhou Vocational Technical College, Chenzhou, Hunan, China

**Keywords:** educational heritage, environmental psychology, environmental-quality evaluation, heritage-related discourse, multimodal user-generated content, selective multimodal representation, visitor appraisal

## Abstract

**Introduction:**

Research on heritage experience has examined material settings, cultural meanings, and visitor appraisal through psychological measures and digital visitor records. Their relationships within the same multimodal records remain less clear.

**Methods:**

This study examines these relationships in educational heritage using 2,855 Ctrip reviews, 4,531 valid sentences, and 6,975 visitor-uploaded images from four Chinese academies, including 829 reviews with matched text and images. Sentence-level topic and valence classification, image classification, review-level association analysis, and coding of explicit evaluations of environmental qualities (EEQE) were combined.

**Results:**

Buildings and cultural symbols accounted for 72.46% of images, while text covered a broader range of heritage, environmental, and practical visit content. Among reviews containing visible heritage imagery, 47.65% also contained heritage-related discourse. None of the 35 topic–image associations met the Benjamini–Hochberg FDR criterion after adjustment for academy, sentence count, and image count. In 617 identical reviews, the association between heritage-related discourse and textual valence weakened after heritage-related sentences were removed; the paired coefficient difference was 0.098 (95% CI 0.049–0.147). Heritage-related discourse showed no statistically reliable association with platform star ratings. EEQE coding identified judgments of atmosphere, tranquility, solemnity, artificiality, and related qualities that were only partly aligned with whole-review valence.

**Discussion:**

The findings show how different media and evaluative indicators preserve complementary aspects of heritage experience. Connecting visual representation, heritage-related discourse, environmental-quality appraisal, textual valence, and ratings within individual reviews provides a naturalistic framework for examining visitor–environment relations while retaining the specific information conveyed by each form of expression.

## 1 Introduction

Educational heritage is experienced through places as well as through history. Visitors to Chinese academies encounter buildings, courtyards, inscriptions, vegetation, other visitors, interpretation services, and practical conditions of access and movement. These elements form a socio-material environment in which historical and educational meanings are encountered during an actual visit. Environmental psychology therefore offers a useful perspective for studying educational heritage. Research on place meaning and place attachment has long shown that physical settings, meanings attributed to places, affective relationships, and behavioral responses are connected but conceptually distinct (Stedman, 2003; Manzo, 2003; Scannell and Gifford, 2010; Lewicka, 2011). Ries and Schwan (2023) similarly describe historically significant places as settings in which environmental characteristics, visitor knowledge, and psychological and social processes interact.

This distinction is important because heritage experience cannot be reduced to the presence of historic features alone. What visitors see in an environment, what they say about its cultural meaning, how they evaluate particular environmental qualities, and how they evaluate the visit as a whole are different aspects of experience. Recent studies illustrate this distinction using direct psychological measures. Ries and Schwan (2024) examined cognitive and affective responses to historical significance; Yang et al. (2024) separated environmental appraisal from emotional response; Fogarasi et al. (2026) measured the connotative meaning of built heritage; and Miola et al. (2026) separately assessed valence, arousal, and perceived restorativeness. Related heritage research has also examined place attachment, cultural identity, perceived value, environmental awareness, embodied cognition, and satisfaction using quantitative and qualitative approaches (Chan et al., 2024; Cai S. et al., 2025; Cai W. et al., 2025; Fu and Dong, 2025; Zhu et al., 2025). Together, this literature suggests that different dimensions of heritage experience should be examined at the level at which they are actually observed or measured.

User-generated content (UGC) provides a complementary way to study these experiences in natural visit settings. Online reviews show what visitors choose to describe and evaluate, while uploaded photographs show what they choose to represent visually. Recent studies have used review text to examine cultural ecosystem services, perceived environmental qualities, and heritage destination image (Zhou and Liu, 2026; Song et al., 2026; Jia et al., 2024; Zang et al., 2025). Research integrating textual, visual, and other digital evidence has examined nature experience, urban environments, heritage tourism, and destination image (Schertz et al., 2026; Wang, 2024; Lin et al., 2026; Du et al., 2026; Hu and Geng, 2024; Su et al., 2025; Zhao and Agyeiwaah, 2025; Tan et al., 2025). This work demonstrates the value of multimodal UGC for studying visitor experience. At the same time, review text and uploaded photographs are selective forms of public expression. They provide different kinds of evidence about a visit and should not automatically be treated as equivalent observations of the same psychological process.

This issue is particularly important in educational heritage settings, where material features and symbolic meanings are closely intertwined. Visitors may photograph buildings, inscriptions, or landscapes, while their written reviews may discuss history, scholarly atmosphere, interpretation, environmental qualities, or practical aspects of the visit. A further distinction exists within textual evaluation: an explicit judgment about a particular environmental quality is not identical to the overall evaluative tone of a review, and neither is equivalent to a platform star rating. Previous studies have examined many of these elements separately, but less is known about how these different forms of representation and evaluation relate within the same naturally occurring multimodal visitor records. Educational heritage therefore provides a useful context for examining how material, cultural, environmental, and evaluative aspects of place experience are expressed across different forms of UGC.

Against this background, this study analyzes Ctrip reviews and visitor-uploaded photographs from Yuelu, Bailudong, Songyang, and Yingtian academies. It addresses three research questions:

(1) What material, cultural, social, and functional aspects of educational-heritage environments are represented in visitors’ review text and uploaded photographs, and what environmental qualities are explicitly evaluated in their reviews?

(2) How do textual and visual representations correspond within the same visitor-generated records when differences among academies and differences in the amount of textual and visual material are taken into account?

(3) How are heritage-related discourse, explicit environmental-quality evaluations, textual valence, and platform star ratings related as different forms of visitor appraisal?

By addressing these questions, the study makes three contributions. First, it distinguishes several forms of digital evidence that are often combined in multimodal heritage research but represent different aspects of person–environment experience. Second, it examines how educational heritage is represented across text and images within the same naturalistic visitor records. Third, it shows how multimodal UGC can complement direct psychological research by providing large-scale evidence of what visitors publicly represent and evaluate after visiting heritage environments.

## 2 Literature review and conceptual framework

### 2.1 Environmental meaning and appraisal in historically significant places

Historically significant places involve several related but distinct aspects of person–environment experience. A material feature can first be encountered as part of the physical setting. It may also acquire historical, cultural, or personal meaning. Visitors may then evaluate particular qualities of the setting or express broader affective responses. These aspects can influence one another, but they refer to different objects of observation and measurement. Research on historically significant places therefore benefits from distinguishing the environment itself from the meanings and evaluations associated with it (Ries and Schwan, 2023; Fogarasi et al., 2026).

This distinction is also evident in studies of visual environmental experience. Different methods capture different aspects of perception. Sherborne and Malthouse (2026), for example, related the presence of historic buildings to crowdsourced scenicness judgments. Fang et al. (2024) assessed streetscape visual quality from the perspective of landscape–perception coupling. Gönül and Durak (2025) compared human judgments of historical streets with deep-learning-based visual assessment. Other studies used eye tracking or physiological sensing to examine how people actually attend to and perceive historical environments (Zhang et al., 2026; Guo and Li, 2025). These approaches all concern environmental experience, but they do not measure the same phenomenon.

For educational heritage, the same principle applies. Buildings, inscriptions, courtyards, and landscapes are environmental features. Their historical or educational meanings represent another level of experience. Judgments such as tranquility, solemnity, or artificiality concern qualities attributed to the setting, while positive or negative affect represents a broader evaluative response. These levels are therefore treated as related but analytically distinct components of place experience.

### 2.2 Heritage-related discourse and interpretation

Heritage interpretation adds a communicative dimension to visitor experience. Interpretation can provide historical information, explain cultural significance, connect material features with broader narratives, and encourage visitors to reflect on heritage values. Experimental research shows that the structure of interpretive information can influence how people connect visual details with meaning (Glaser et al., 2022). Heritage-tourism studies have also examined how interpretation content relates to cultural inheritance, cultural identity, perceived responsibility, and preservation intentions (Ruan et al., 2024; Wang M. Y. et al., 2025).

Interpretive outcomes can also depend on the medium through which information is delivered. Zheng et al. (2024) compared different interpretation media in environmental education, while Wang H. et al. (2025) examined how heritage interpretation messages relate to perceived heritage value, environmental sensitivity, and pro-environmental behavior. These studies illustrate that interpretation can be studied as an intervention or communication process when exposure and psychological outcomes are measured directly.

Visitor-generated reviews record the cultural and interpretive content that visitors choose to mention after their visit. In the present framework, heritage-related discourse refers to textual references to historical-cultural content, scholarly atmosphere, and interpretation- or knowledge-related experience. Such discourse may contain appreciation, criticism, description, or reflection. It records public expression about heritage. Cultural understanding concerns visitors’ comprehension of heritage significance, and perceived heritage value concerns the importance or worth they attribute to it; investigating these outcomes requires corresponding measures of understanding and value.

A stronger assessment of whether visitor discourse reproduces institutional meanings requires an external reference. Du et al. (2026), for example, compared public multimodal expression with a professional-intention corpus in industrial-heritage regeneration. Such comparisons make it possible to examine convergence and divergence between institutional and public narratives. More generally, this distinction helps separate what heritage institutions communicate from what visitors subsequently choose to express.

Heritage communication also depends on organizational and destination conditions. Yao et al. (2026) examine digital marketing and sustainable performance in cultural heritage tourism, while Yao et al. (2024) examine coordination between the MICE and tourism industries. These perspectives place communication within broader management and resource relationships. For the present study, they provide a context for considering how visitor feedback could inform heritage management; direct measures of organizational practices would be needed to explain how those practices shape individual visitor expression.

### 2.3 Environmental-quality evaluation and broader evaluative indicators

Environmental appraisal becomes more informative when the object of evaluation is retained. Visitors may evaluate accessibility, tranquility, attractiveness, usability, atmosphere, maintenance, or other specific qualities of a setting. Song et al. (2026) showed that environmental perception in park reviews can be represented through several dimensions. Mohamed and Kronenberg (2025) similarly distinguished accessibility from attractiveness and identified practical concerns such as fees and maintenance in online reviews. These studies demonstrate the value of identifying what aspect of the environment is being evaluated.

Object-specific environmental evaluation differs from broader textual evaluation. A review may criticize one feature of a place while remaining positive overall, or it may praise a particular environmental quality while expressing dissatisfaction with other aspects of the visit. Research that combines different data sources also supports this distinction. Liao et al. (2026) used UGC for exploratory analysis and separate survey measures for appraisal, awe, restoration, and word-of-mouth. Lai and Deal (2025) likewise showed that online expression and other measures of environmental perception can provide different forms of evidence.

Platform ratings represent another evaluative level. A star rating compresses a visit into a small number of ordered categories, whereas review text can contain several objects of praise, criticism, or ambivalence (Bigne et al., 2023). Research on online reviews has documented mismatches between textual sentiment and numerical ratings (Sugathan et al., 2024). Related studies have treated review inconsistency and rating inconsistency as substantive phenomena (Wang D. et al., 2025; Wang P. et al., 2025).

These distinctions provide the conceptual basis for separating three forms of evaluation in the present framework: explicit evaluation of particular environmental qualities, overall evaluative language in review text, and platform star ratings. They may be related, but each preserves a different level of information about visitor appraisal.

### 2.4 Multimodal UGC as selective naturalistic evidence

Text and images differ in what they can readily express. Photographs are well-suited to visible features such as buildings, inscriptions, landscape elements, and human activity. Text can more readily express historical relations, atmosphere, interpretation experience, practical problems, and explicit judgments. Multimodal UGC therefore provides several views of the same visit. Research comparing social-media imagery with more systematic environmental records has demonstrated this selectivity (Wang, 2024). Tan et al. (2025) examined how combinations of visual, textual, and audio content relate to user engagement, while Zhan et al. (2024) provided a methodological review of image analytics in tourism and hospitality.

Selection is central to the interpretation of visitor-uploaded photographs. An uploaded image records what a visitor chose to photograph and share. It does not directly record all environmental features that were available during the visit. Nor does image presence alone indicate the amount of visual attention given to a feature. Direct visual-perception studies use different evidence. Zhang et al. (2026) measured fixation patterns with eye tracking; Guo and Li (2025) combined eye-tracking and physiological data; Gönül and Durak (2025) compared human perception with computational visual assessment; and Fang et al. (2024) focused on visual aesthetic quality. Crowdsourced scenicness judgments provide another form of environmental evaluation (Sherborne and Malthouse, 2026). Computational image categories also require context-specific validation (Nixon, 2024).

The same distinction applies to multimodal heritage research. Du et al. (2026) introduced a professional-intention corpus to provide an external reference for public expression. Other multimodal studies demonstrate that text and imagery can be analyzed together, but their relationship must be interpreted in light of how each modality is produced and selected. Platform context also matters. Guo et al. (2021) found differences in destination-image representation across five Chinese travel-review platforms. Comparisons across platforms therefore need to consider user populations, posting formats, and the availability of comparable content.

Cross-modal correspondence in UGC is therefore best understood as a relationship between different forms of visitor-selected public expression. It can reveal whether certain textual and visual contents tend to appear together, while preserving the different evidential meanings of the two modalities.

### 2.5 Conceptual synthesis: observational layers in multimodal educational-heritage UGC

Drawing on the preceding literature, the conceptual framework distinguishes five observational layers in multimodal educational-heritage UGC: visitor-selected visual content, heritage-related discourse, explicit environmental-quality evaluation, textual valence, and platform star ratings.

These layers can occur within the same visitor record, but they describe different aspects of experience. Visual content records what visitors choose to show. Heritage-related discourse records what cultural and interpretive content they choose to discuss. Explicit environmental-quality evaluation retains the specific environmental object or quality being judged. Textual valence summarizes the broader evaluative direction of expressed language. Platform ratings provide a structured overall judgment.

Figure 1 summarizes these five layers and their review-level relationships. The framework treats them as connected observations with different measurement targets. This structure provides the conceptual basis for the research questions introduced in Section “1 Introduction” and for the analyses that follow.

**FIGURE 1:**
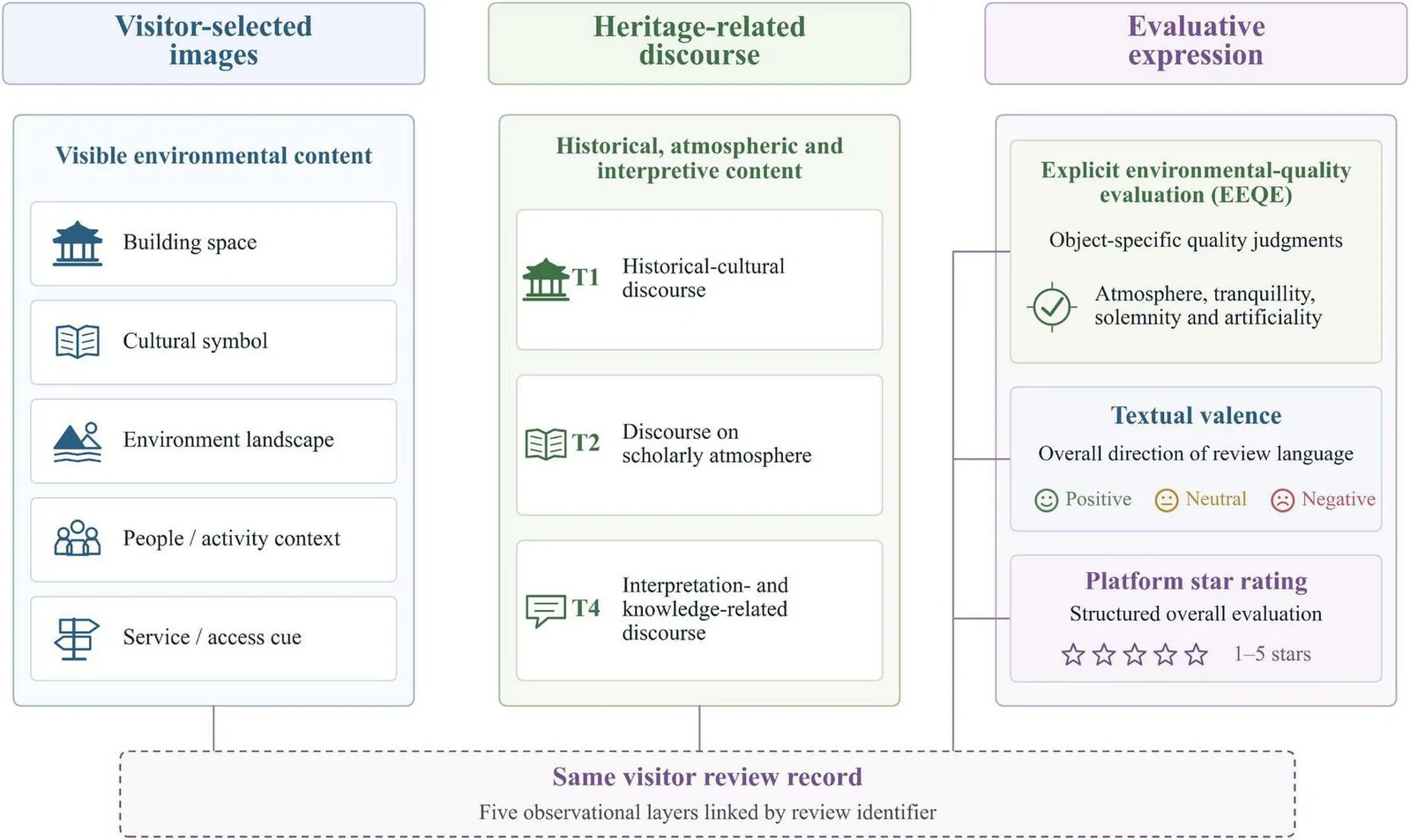
Observational layers in multimodal educational-heritage visitor records. Five linked observations distinguish visitor-selected image content, heritage-related discourse, explicit environmental-quality evaluation, textual valence, and platform star ratings. Lines connect observations to the same review record and do not indicate a temporal sequence.

## 3 Materials and methods

### 3.1 Study sites, data, and analytical design

This study analyzed publicly available Ctrip reviews and visitor-uploaded photographs from four Chinese academies: Yuelu, Bailudong, Songyang, and Yingtian. Yuelu, Bailudong, and Songyang constituted the core comparative sites. Yingtian was included in the common classification framework and used mainly for descriptive analysis because its available corpus was substantially smaller. Songyang Academy forms part of the UNESCO World Heritage property Historic Monuments of Dengfeng in “The Centre of Heaven and Earth,” while Bailudong Academy is located within the broader Mount Lushan cultural landscape (UNESCO World Heritage Centre, 2026a,b). The four academies share an educational-heritage function but differ in historical development, physical setting, and contemporary tourism context. Academy was therefore included as a site-level covariate in the multivariable analyses. The locations of the four academies are shown in Figure 2.

**FIGURE 2:**
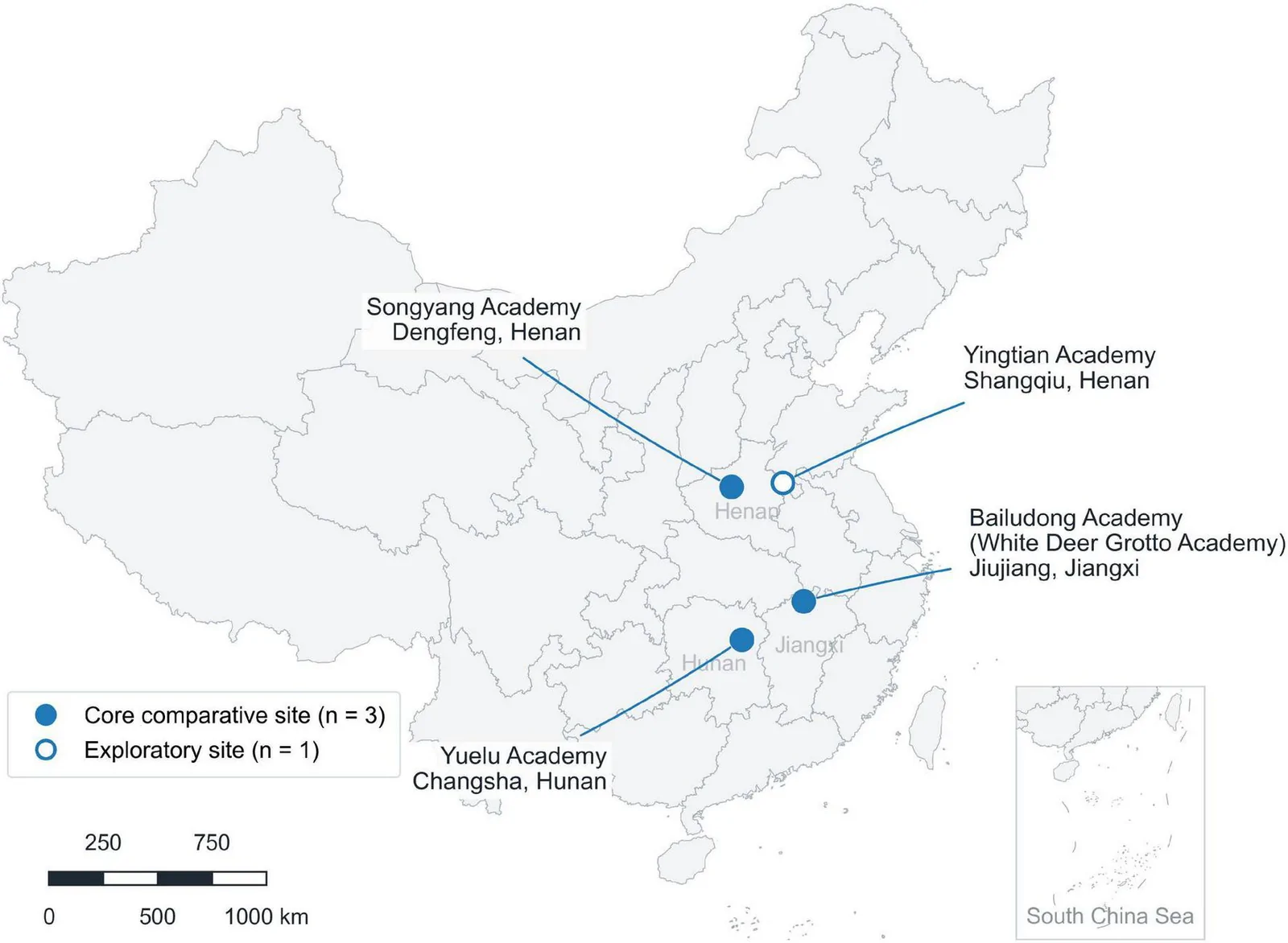
Locations of the four Chinese academies. Filled circles identify the core sites: Yuelu in Changsha, Hunan; Bailudong near Jiujiang, Jiangxi; and Songyang in Dengfeng, Henan. The open circle identifies Yingtian in Shangqiu, Henan, included as an exploratory site. Coordinates are approximate site locations. The map was created by the authors using Python, with basemap data obtained from Tianditu (https://www.tianditu.gov.cn/).

Reviews and associated photographs were retrieved on 28 February 2026. Recorded review dates ranged from March 2016 to February 2026. Data processing included duplicate removal, exclusion of irrelevant or low-information text, sentence segmentation, image-quality screening, and linkage of text and images by review ID. The final corpus comprised 2,855 valid reviews, 4,531 valid sentences, and 6,975 valid images. Of the reviews, 829 contained both valid text and valid images and formed the matched multimodal sample. A nested core subset of 812 matched reviews came from Yuelu, Bailudong, and Songyang. Corpus coverage and analytical sample definitions are summarized in Table 1 and Supplementary Tables 1–3.

**TABLE 1:** Corpus coverage and analytical samples.

Academy	Role	Valid reviews	Valid sentences	Valid images	Matched reviews	Visible-heritage reviews	Dominant visual category
Yuelu	Core	1,817	2,780	3,851	478	446	389
Bailudong	Core	459	765	1,479	163	159	129
Songyang	Core	523	864	1,484	171	165	124
Yingtian	Exploratory	56	122	161	17	17	13
Total	–	2,855	4,531	6,975	829	787	655

Valid reviews denotes the 2,855-review corpus; sentence predictions cover 2,854 reviews. Visible-heritage reviews contain at least one image classified as Building space, Cultural symbol, or Environment landscape. Dominant visual category counts reviews with a single most frequent image category; ties remain distinct in regression coding. Yingtian is exploratory.

Sentence-level units were used for topic and valence classification, image-level units for visual classification, and review-level units for multimodal aggregation and statistical analysis. Ctrip provided linked text, photographs, and star ratings within a common posting environment. The study examines relationships among these forms of expression within individual review records. The analyses characterize the retrieved Ctrip corpus, with the matched subset defined by the availability of both valid text and valid photographs.

### 3.2 Text analysis, classification, and human validation

The textual topic framework was developed from reviews of the three core academies and then applied to the full sentence corpus. Topic discovery used a balanced sample from the three sites. A total of 346 eligible reviews were sampled without replacement from each academy. Text preprocessing yielded 1,037 non-empty discovery documents. This sample was used to derive the topic structure, whereas sentence-level classification and subsequent review-level analyses used the full eligible corpus. Corpus coverage, topic definitions, and model settings are summarized in Supplementary Tables 1, 2, 4, 5.

Topic discovery used paraphrase-multilingual-MiniLM-L12-v2 embeddings with BERTopic and KMeans (Reimers and Gurevych, 2019; Grootendorst, 2022). Seven categories were retained to distinguish historical-cultural references, scholarly atmosphere, architectural form, interpretation and knowledge, environmental experience, service and access, and the course of the visit. Comparisons of the same 1,037 discovery documents at k = 5–8 helped assess this level of detail. At k = 6, one cluster combined 117 reviews from the retained historical-cultural category and 116 from the scholarly-atmosphere category; the seven-category solution kept these contents separately available for analysis. The k = 8 solution introduced further subdivisions of environmental and visit-process content. A supplementary comparison on 4,217 unique core-site sentences assessed clustering at k = 5–8 using silhouette, Calinski–Harabasz, and Davies–Bouldin indices. The criteria favored different resolutions. Seven categories were therefore retained on substantive grounds, with the original review-level comparisons and the separate sentence-level diagnostics reported in Supplementary Tables 6, 7.

The seven categories were: T1, Historical-cultural discourse; T2, Discourse on scholarly atmosphere; T3, Architectural heritage and spatial form; T4, Interpretation- and knowledge-related discourse; T5, Environmental landscape and leisure experience; T6, Service- and access-related discourse; and T7, On-site process and localized experience. A prototype embedding was constructed for each category from its discovery documents. Each cleaned sentence was assigned to the category with the highest cosine similarity. Review-level topic presence was then derived from all sentence assignments within the same review, allowing multi-sentence reviews to contain more than one topic. Operational definitions of the seven categories are provided in Supplementary Table 4. Illustrative excerpts are provided in Supplementary Table 8.

The final sentence-level topic assignments were evaluated using a stratified human-validation sample. Sampling was stratified by academy and assigned topic across the non-empty academy-by-topic strata and yielded 300 sentences from 279 reviews. Two authors independently coded each sentence using the fixed seven-category codebook. The assigned topic was concealed during independent coding, while surrounding review context was available when needed for interpretation. Disagreements were resolved through consensus adjudication. Inter-coder reliability and agreement between the sentence-level assignments and adjudicated human labels are reported in Table 2 and Supplementary Table 9.

**TABLE 2:** Classification methods and validation.

Module	Model and classification	Settings and analytical unit	Validation
Text topics	Paraphrase-multilingual-MiniLM-L12-v2 + BERTopic/KMeans; prototype-based sentence projection	1,038 sampled reviews → 1,037 discovery documents; seven retained topics; highest cosine-similarity sentence assignment; no abstention threshold	Two-author validation, *N* = 300 sentences: Cohen’s kappa = 0.687; adjudicated top-1 accuracy = 0.793. The validation design and summary are reported in Supplementary Table 9.
Sentiment	hfl/chinese-roberta-wwm-ext; negative/neutral/positive classification	Maximum length = 128; learning rate = 2 × 10^–5^; batch sizes = 16/32; class-weighted loss; up to eight epochs; validation macro-F1 early stopping	Review-disjoint train/validation/test: 490/105/105 sentences; 448/96/97 reviews. Test accuracy = 0.8952; macro-F1 = 0.8858; zero review overlap (Supplementary Tables 10–12).
Image categories	openai/clip-vit-base-patch32 + logistic regression	512-dimensional L2-normalized embeddings; StandardScaler; C = 4.0; class_weight = balanced; max_iter = 5000. High confidence: score ≥ 0.80, margin ≥ 0.30, no review flag.	Held-out *N* = 215: accuracy = 0.800; balanced accuracy = 0.771; macro-F1 = 0.769; weighted-F1 = 0.800; top-2 accuracy = 0.949 (Supplementary Tables 13, 14).

Topic validation used two authors’ independent coding of 300 stratified sentences (κ = 0.687; adjudicated top-1 accuracy = 0.793). Sentiment performance refers to the review-disjoint test set. Image performance refers to the held-out estimator; the final projection estimator was fitted to all 1,043 reference images. All 6,975 image labels enter the main analyses. Review flags identify a top-1 score < 0.60 or a top-1/top-2 margin < 0.15. Further settings and category-level validation are provided in the Supplementary material.

Sentence-level evaluative valence was classified as negative, neutral, or positive using a fine-tuned hfl/chinese-roberta-wwm-ext model (Cui et al., 2020). The human-labeled reference set contained 700 sentences from 641 reviews, combining an initial 200-sentence pilot with 500 additional labeled sentences. The pilot material was independently coded by the two researchers, and disagreements were adjudicated before the reference labels were finalized. The full reference set was partitioned by review ID into training, validation, and test sets of 490, 105, and 105 sentences, respectively, with no review shared across partitions. Model training used class-weighted cross-entropy and validation-based early stopping. The data split, classification performance, and confusion matrix are reported in Supplementary Tables 10–12; the principal model settings and validation summary are provided in Table 2.

### 3.3 Image classification and validation

Images were classified into five mutually exclusive visual categories: Building space, Cultural symbol, Environment landscape, People/activity context, and Service/access cue. Mixed or ambiguous images identified during reference-set review were excluded from development of the five-class classifier.

One author manually assigned and reviewed the image reference labels using the five-category definitions. The final reference set comprised 1,043 images: 818 retained labels and 225 eligible labels from a 240-image review set. Fifteen images with mixed or ambiguous content were excluded from that review set. Images were converted to RGB and processed using the AutoProcessor associated with openai/clip-vit-base-patch32 (Radford et al., 2021). The resulting 512-dimensional CLIP embeddings were L2-normalized and standardized before logistic regression. The classifier used C = 4.0, balanced class weights, the lbfgs solver, and a maximum of 5,000 iterations. Principal settings and reference-set details are reported in Table 2 and Supplementary Table 15.

Validation used a review-group-aware split so that images originating from the same review could not occur in both the training and validation sets. A stratified group split by review ID yielded 828 training images and 215 held-out validation images. Classification performance was evaluated using accuracy, balanced accuracy, macro-F1, weighted-F1, and top-2 accuracy. Per-category validation metrics and the confusion matrix are reported in Supplementary Tables 13, 14. After validation, the classifier was fitted to all 1,043 reference images and applied to the 6,975 valid images.

All 6,975 valid images retained their highest-scoring category in the primary analysis. For the sensitivity analysis, 0.80 was used as a selective cutoff on the classifier’s highest category score. Together with a top-1/top-2 score margin of at least 0.30 and the absence of a review flag, this rule identifies images for which the classifier assigns a clear preference to one category. Review flags were defined by a top-1 score below 0.60 or a score margin below 0.15. The 0.80 rule retained 6,307 images (90.42%), providing a more selective subset while preserving broad corpus coverage. Retention at score cutoffs of 0.60, 0.70, and 0.90 was 96.56%, 93.68%, and 85.02%, respectively (Supplementary Table 15). The score describes model confidence; held-out accuracy is reported separately in Supplementary Tables 13, 14. Category-specific associations under the high-confidence rule are reported in Supplementary Table 16.

### 3.4 Review-level measures and environmental-quality coding

Sentence-level topic assignments, sentence-level valence predictions, and image categories were aggregated by review ID. A textual topic or visual category was coded as present when it occurred at least once within a review. Building space, Cultural symbol, and Environment landscape represented visible material or symbolic environmental content. People/activity context represented visible social activity, whereas Service/access cue represented visible functional or service-related content. Definitions of the review-level observational indicators are summarized in Supplementary Table 17.

For review-level analyses, heritage-related discourse was coded as present when at least one sentence belonged to the historical-cultural (T1), scholarly-atmosphere (T2), or interpretation/knowledge (T4) category. The three component categories were also retained separately for component-specific analyses. Interpretation/knowledge discourse included requests for explanation, evaluations of guiding or knowledge communication, and statements explicitly describing learning or understanding.

Review-level textual valence was calculated as


$${\text{V}}_{\text{i}}\,\text{ = }\,\frac{{\text{n}}_{\text{positive}}\,{\text{-n}}_{\text{negative}}}{\text{n}}$$

where n was the number of classified sentences in review i. Alternative valence summaries included the mean sentence-level difference between predicted positive and negative probabilities and the proportion of positive sentences. Platform star ratings were retained in their original 1–5 form and analyzed as a separate evaluative indicator.

Guided by previous research that distinguishes object-specific environmental appraisal from broader affective and overall evaluative measures (Yang et al., 2024; Song et al., 2026; Mohamed and Kronenberg, 2025), we developed a content-coding measure of explicit evaluations of environmental qualities (EEQE). An eligible statement explicitly attributed a quality to the academy or its immediate setting. The codebook covered historical or academic atmosphere, tranquility or serenity, solemnity or depth, explicitly restorative-like wording, disturbance or artificiality, and other specified environmental qualities. EEQE presence and evaluative direction were coded separately. Factual historical descriptions and lists of environmental objects were coded as EEQE only when they also contained an explicit attribution of environmental quality.

The EEQE coding sample comprised 700 reviews. A rule-based screening procedure was first used to identify core-site reviews that were more likely to contain explicit environmental-quality expressions. The screening framework covered five principal candidate dimensions: historical/academic atmosphere, tranquility/serenity, explicit restorative-like expression, solemnity/depth, and disturbance/artificiality. The documented screening anchors available for these dimensions are summarized in Supplementary Table 18. The screening step determined candidate status only; final EEQE labels were assigned through author coding.

The 700-review coding sample included 419 core-site candidate reviews identified by the screening procedure, 240 stratified core-site reviews that were not identified by the screen (80 from each core academy), and 41 Yingtian reviews. The 240 screen-unidentified reviews provided a comparison sample for evaluating how much EEQE content was missed by the candidate-screening procedure.

Two authors independently coded all 700 reviews using the same EEQE codebook. Disagreements were resolved through adjudication. Pre-adjudication inter-coder reliability was evaluated on 697 comparable coding pairs. For binary presence analyses, present and mixed were coded as presence, absent as absence, and unclear as missing. The EEQE coding design and inter-coder reliability are reported in Supplementary Table 19. Adjudicated label distributions and cross-classifications with adjacent textual indicators are reported in Supplementary Table 20. Screening coverage, false-omission estimates, and estimated recall are reported in Supplementary Table 21, while the documented candidate-screening anchors are listed in Supplementary Table 18.

### 3.5 Cross-modal correspondence and robustness analyses

Cross-modal correspondence was examined at the review level in the 829 reviews containing both valid text and valid images. The seven textual topic-presence indicators were crossed with the five visual category-presence indicators, yielding 35 topic–visual combinations. Bivariate associations were summarized using 2 × 2 co-occurrence counts, Haldane–Anscombe corrected odds ratios with 95% confidence intervals, Lift, Phi, and two-sided Fisher exact tests. Benjamini–Hochberg correction was applied across the complete family of 35 tests (Benjamini and Hochberg, 1995). The full bivariate association matrix is reported in Supplementary Table 22.

Covariate-controlled logistic models were then used to account for site differences and variation in the amount of textual and visual material available within each review. For each topic–visual combination, visual-category presence was modeled as a function of textual-topic presence, academy, log(1+valid sentence count), and log(1+valid image count). Huber–White robust standard errors were used, and the resulting 35 *p*-values were corrected as one Benjamini–Hochberg family. The complete covariate-controlled matrix is reported in Supplementary Table 23. The 829 matched reviews constituted the primary multimodal sample, while the 812 matched reviews from the three core academies were used as a nested sensitivity sample; the corresponding sample comparison is summarized in Supplementary Table 24. The complete bivariate matrices for both samples are provided in Supplementary Table 25.

Review-level concurrence between visible heritage content and heritage-related discourse was examined separately from the 35 pairwise analyses. Visible heritage content was defined as the presence of at least one Building space, Cultural symbol, or Environment landscape image. Heritage-related discourse was defined as the presence of historical-cultural, scholarly-atmosphere, or interpretation/knowledge discourse within the same review. Conditional concurrence proportions were estimated with Wilson 95% confidence intervals (Wilson, 1927) and compared between single-sentence and multi-sentence reviews. The complete single- versus multi-sentence comparison is reported in Supplementary Table 26. Related topic combinations, overlap counts, and site-model specifications are reported in Supplementary Tables 27–31.

A separate visual-confidence sensitivity analysis used the high-confidence image labels defined in Section “3.3 Image classification and validation.” For each visual category, presence was coded as 1 when at least one high-confidence image belonged to that category. Absence was assigned only when all images linked to the review were high confidence and none belonged to that category; otherwise, the category was treated as unresolved for that review. The same covariates and multiple-testing procedure were then applied, using log(1+high-confidence image count) as the image-volume covariate. Confidence-group information is reported in Supplementary Table 15, and the category-specific high-confidence association results are reported in Supplementary Table 16.

### 3.6 Evaluative association analyses

Associations between heritage-related discourse and review-level textual valence were estimated in the 812 core matched reviews using ordinary least squares with HC3 robust standard errors (Long and Ervin, 2000). The main model included heritage-related discourse, service/access-related discourse, review length, image count, academy, dominant or tied visual category, and review period. Component-specific models replaced the combined heritage-related-discourse indicator with the historical-cultural, scholarly-atmosphere, and interpretation/knowledge categories individually and jointly. The joint model was evaluated with a Wald test, followed by pairwise coefficient contrasts. Main and component-specific specifications are reported in Supplementary Tables 32–34. A secondary model examined the interaction between heritage-related discourse and service/access-related discourse; these models are reported in Supplementary Table 35.

A within-review coefficient-difference analysis was conducted in the 617 core matched reviews containing at least one sentence outside the heritage-related discourse categories. Complete-review valence and remaining-text valence were estimated with the same predictors and covariates. The within-review difference between the two valence outcomes was then modeled using the same design matrix and HC3 robust standard errors. The heritage-related-discourse coefficient in this model estimates the paired difference between its coefficients in the two outcome models. The complete- and remaining-text specifications and the formal coefficient-difference analysis are reported in Supplementary Table 36.

Platform star ratings were analyzed in the 2,798 core reviews with sentence-linked text. An ordered logistic model was first specified for the original 1–5 rating scale, with heritage-related discourse, service/access-related discourse, sentence count, academy, and review period as predictors. The proportional-odds assumption was assessed using parallel-lines diagnostics. When the common-slope assumption was not met, threshold-specific cumulative-logit models were estimated at the four native cut points (rating > 1, rating > 2, rating > 3, and rating > 4) using the same covariates and review-clustered robust standard errors. An HC3 OLS model treating the 1–5 rating as numeric was used as a scale-sensitivity analysis. Rating-model diagnostics and threshold-specific specifications are reported in Supplementary Table 37. Additional topic and site-interaction results are reported in Supplementary Tables 38, 39.

Two additional sensitivity analyses addressed repeated wording and multimodal-sample selection. First, exact and near-duplicate heritage-related sentences occurring across different reviews of the same academy were identified after text normalization. Flagged sentences were excluded only from a sensitivity version of the heritage-related-discourse indicator. The matching rules, sentence-level flags, and resulting review-level indicator changes are reported in Supplementary Table 40. Second, the 812 matched reviews were compared with all 2,798 core sentence-linked reviews using descriptive statistics and a common text-only model. This sample-selection comparison is reported in Supplementary Table 41. Supplementary Table 42 reports the additional within-review sentence-order analysis.

## 4 Results

### 4.1 Corpus characteristics and textual and visual representation

The final corpus comprised 2,855 valid reviews, 4,531 valid sentences, and 6,975 valid images. Cross-modal analyses used 829 reviews containing both valid text and valid images. Of these, 812 reviews from Yuelu, Bailudong, and Songyang formed the nested core sample. Among the core matched reviews, 617 contained at least one sentence outside the heritage-related discourse categories and were eligible for the remaining-text analysis (see Section 3.6).

The 300-sentence human-validation sample yielded Cohen’s κ = 0.687 between the two authors. The final sentence-topic assignments achieved an accuracy of 0.793 against the adjudicated human labels (Table 2 and Supplementary Table 9).

Across the 4,531 sentences, T3 Architectural heritage and spatial form was the most frequent topic, with 1,283 sentences (28.32%). It was followed by T7 On-site process and localized experience (15.40%), T5 Environmental landscape and leisure experience (13.53%), T4 Interpretation- and knowledge-related discourse (13.18%), T6 Service- and access-related discourse (12.60%), T1 Historical-cultural discourse (10.04%), and T2 Discourse on scholarly atmosphere (6.93%). The valence classifier assigned 62.33% of sentences as positive, 25.84% as neutral, and 11.83% as negative. T6 Service- and access-related discourse was the only topic in which negative sentences were more frequent than positive sentences (52.01% vs. 28.55%) (Figure 3 and Table 3).

**FIGURE 3:**
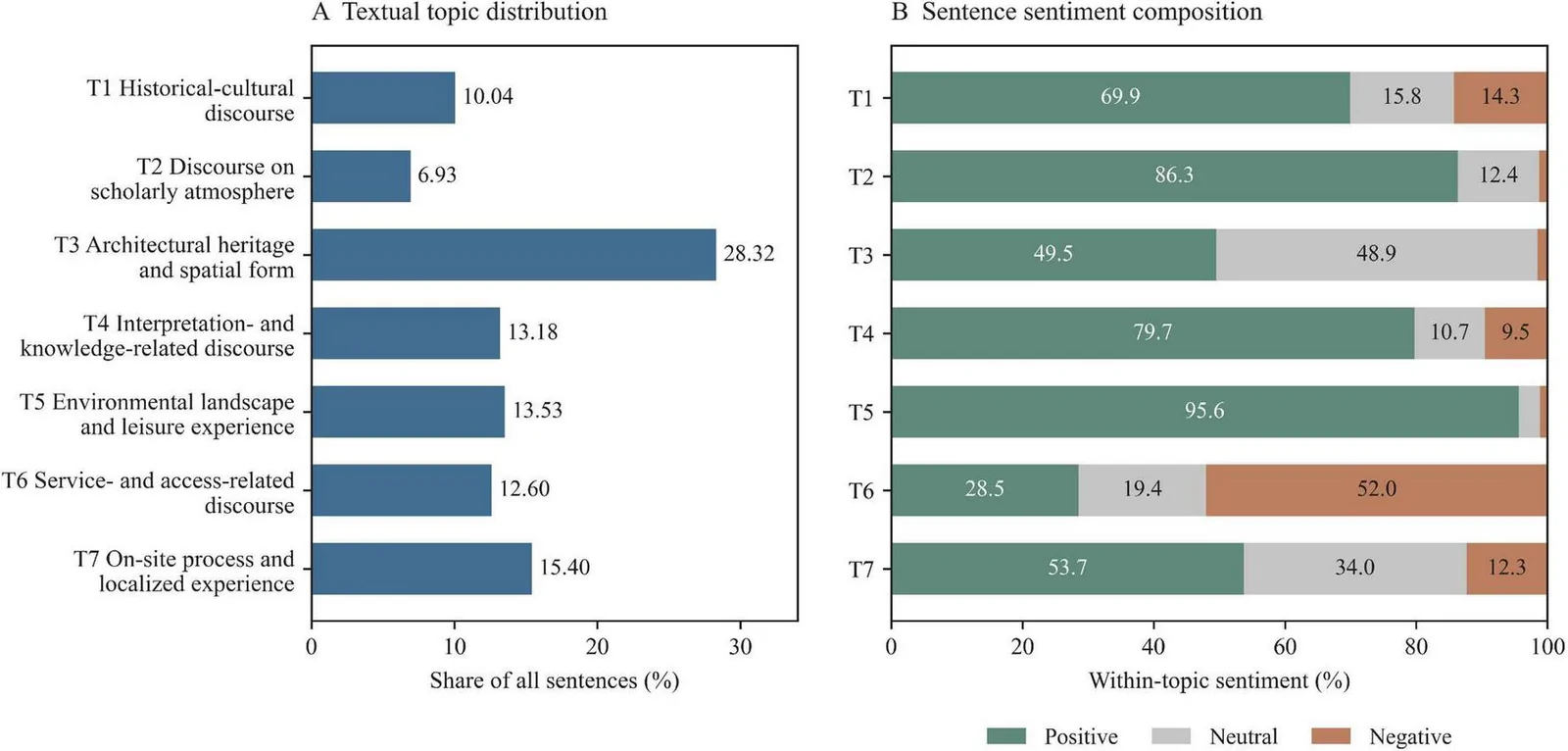
Textual topic share and sentence sentiment composition. **(A)** Shows the percentage of the 4,531 valid sentences assigned to each primary topic. **(B)** Shows the within-topic percentages classified as positive, neutral, and negative by the sentiment classifier trained and evaluated with review-grouped splits. Sentiment categories describe model predictions for expressed text. T1, historical-cultural discourse; T2, discourse on scholarly atmosphere; T3, architectural heritage and spatial form; T4, interpretation- and knowledge-related discourse; T5, environmental landscape and leisure experience; T6, service- and access-related discourse; and T7, on-site process and localized experience.

**TABLE 3:** Textual topic distribution and within-topic sentiment composition.

Topic	Sentences	Share	Positive	Neutral	Negative
T1 historical-cultural discourse	455	10.04%	69.89%	15.82%	14.29%
T2 discourse on scholarly atmosphere	314	6.93%	86.31%	12.42%	1.27%
T3 architectural heritage and spatial form	1,283	28.32%	49.49%	48.95%	1.56%
T4 interpretation- and knowledge-related discourse	597	13.18%	79.73%	10.72%	9.55%
T5 environmental landscape and leisure experience	613	13.53%	95.60%	3.26%	1.14%
T6 service- and access-related discourse	571	12.60%	28.55%	19.44%	52.01%
T7 on-site process and localized experience	698	15.40%	53.72%	33.95%	12.32%

Shares use all 4,531 sentences as the denominator. Positive, neutral, and negative denote within-topic proportions of predicted sentence sentiment.

The visual corpus was more concentrated. Building space accounted for 2,822 images (40.46%) and Cultural symbol for 2,232 images (32.00%), together representing 72.46% of all 6,975 images. Environment landscape accounted for 14.64%, People/activity context for 8.62%, and Service/access cue for 4.29% (Figure 4 and Table 4).

**FIGURE 4:**
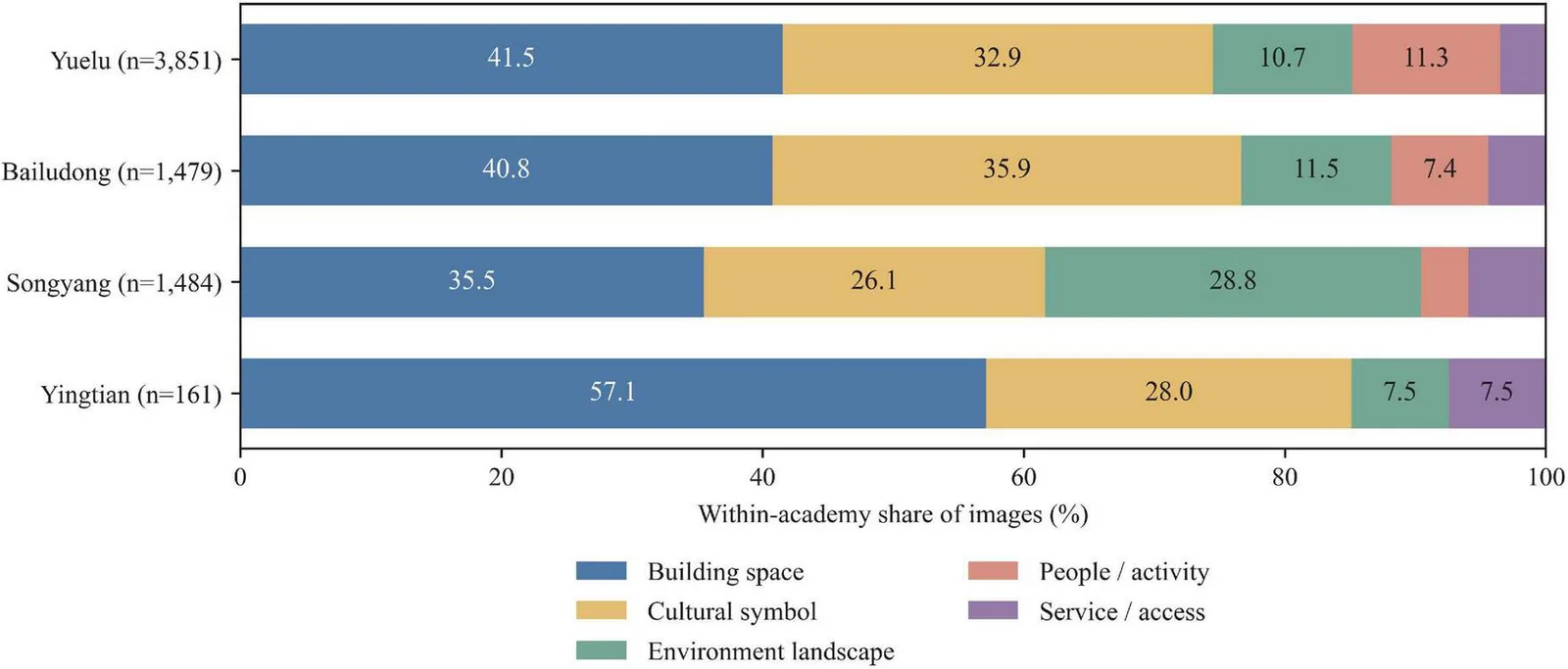
Within-academy composition of five visual categories across 6,975 visitor-uploaded images. Each row sums to approximately 100%, subject to rounding, and percentages use the image total for that academy: Yuelu, 3,851; Bailudong, 1,479; Songyang, 1,484; and Yingtian, 161. Each image contributes its assigned primary visual category. Yingtian is the exploratory site. Percentages describe uploaded images within each academy.

**TABLE 4:** Visual-category distribution across all valid images.

Visual category	N images	Share
Building space	2,822	40.46%
Cultural symbol	2,232	32.00%
Environment landscape	1,021	14.64%
People/activity context	601	8.62%
Service/access cue	299	4.29%

All percentages use 6,975 images as the denominator and may not sum to exactly 100% because of rounding. People/activity context indicates visible people or activity and does not identify formal interpretation.

### 4.2 Cross-modal correspondence and sensitivity analyses

In the bivariate analyses, four of the 35 topic–image combinations met the Benjamini–Hochberg false-discovery-rate criterion (q < 0.05). T3 Architectural/spatial discourse was positively associated with Building space images (OR = 2.57, 95% CI [1.83, 3.63], Lift = 1.117, Phi = 0.191, q < 0.001). T4 Interpretation/knowledge discourse was negatively associated with Building space (OR = 0.47, 95% CI [0.33, 0.67], q < 0.001) and Environment landscape (OR = 0.44, 95% CI [0.30, 0.63], q < 0.001), and positively associated with People/activity context (OR = 1.76, 95% CI [1.25, 2.49], q = 0.015). The complete bivariate matrix is reported in Supplementary Table 22.

In the covariate-controlled models, none of the 35 topic–image associations met the BH-FDR criterion. The association between T3 Architectural/spatial discourse and Building space was OR = 1.55 (95% CI [1.02, 2.36], *p* = 0.042, q = 0.247). The estimate for T4 Interpretation/knowledge discourse and People/activity context was OR = 1.50 (95% CI [1.03, 2.17], *p* = 0.033, q = 0.229). The corresponding estimates for T4 with Building space and Environment landscape were OR = 0.84 (95% CI [0.54, 1.30], *p* = 0.428, q = 0.608) and OR = 0.67 (95% CI [0.44, 1.01], *p* = 0.057, q = 0.247), respectively (Figure 5 and Table 5). The complete covariate-controlled matrix is reported in Supplementary Table 23.

**FIGURE 5:**
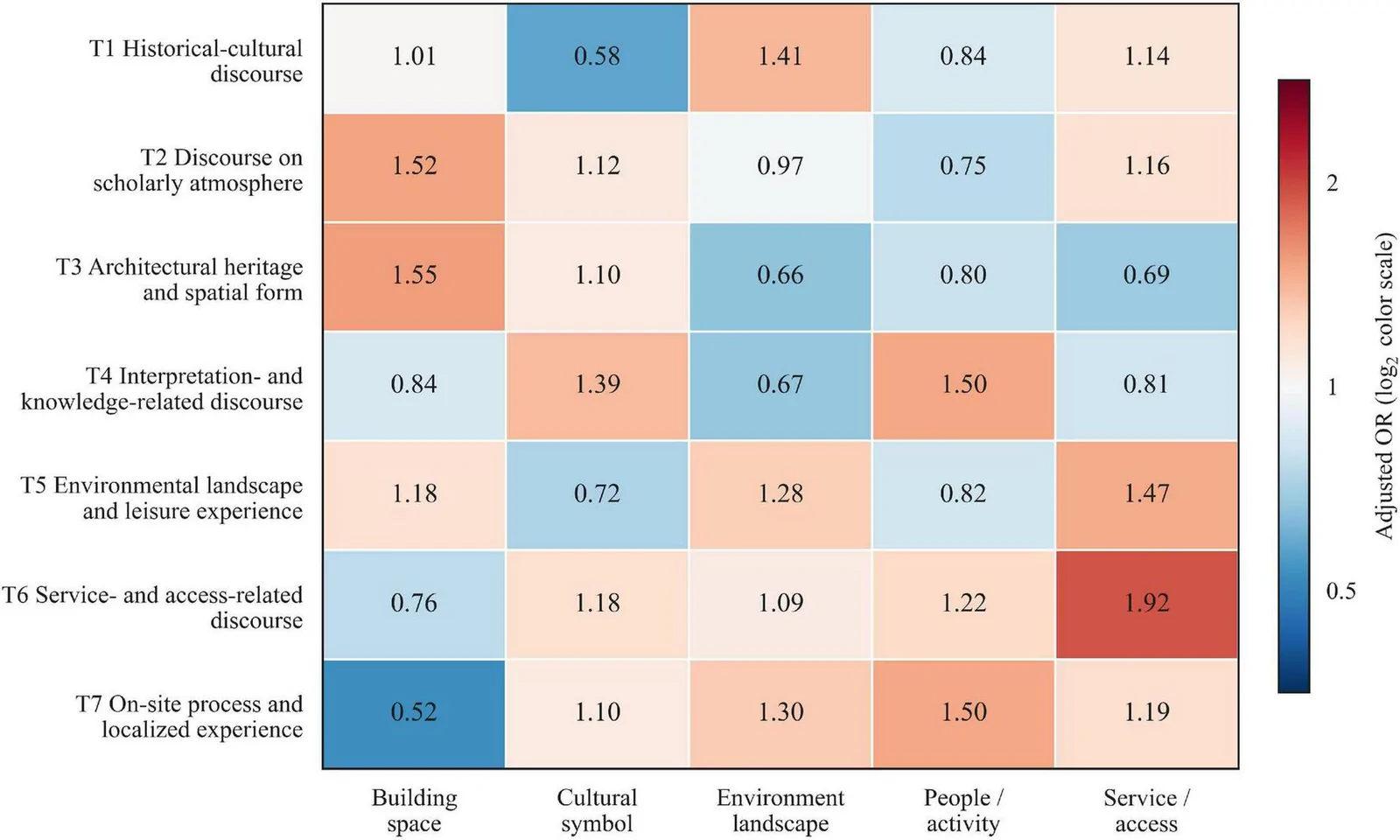
Adjusted topic–image associations in 829 matched reviews. Cells show odds ratios for the 35 topic–visual presence combinations, with color scaled to log_2_(OR). All models adjust for academy, log(1+valid sentence count), and log(1+valid image count), using Huber–White robust standard errors. Benjamini–Hochberg correction was applied to the full 35-test family; no adjusted association met q < 0.05. Complete estimates and confidence intervals are provided in the Supplementary materials.

**TABLE 5:** Bivariate and adjusted associations for four topic–image combinations.

Topic × image	Unadj. OR	Unadj. q	Adj. OR	Adj. 95% CI	Adj. p	Adj. q
T3 × Building space	2.57	<0.001	1.55	1.02–2.36	0.042	0.247
T4 × Building space	0.47	<0.001	0.84	0.54–1.30	0.428	0.608
T4 × Environment landscape	0.44	<0.001	0.67	0.44–1.01	0.057	0.247
T4 × People/activity context	1.76	0.015	1.50	1.03–2.17	0.033	0.229

The four rows are the combinations that met q < 0.05 in the bivariate 35-test family. Adjusted models used the same specification for every combination: academy, log(1+valid sentence count), and log(1+valid image count), with Huber–White robust standard errors; the 35 adjusted *p*-values were again Benjamini-Hochberg corrected. None of the 35 adjusted associations met q < 0.05. Full matrices and nested-sample sensitivity results are reported in the Supplementary material. Bivariate odds ratios and confidence intervals use a 0.5 correction in all four contingency-table cells.

Results from the nested 812-review core sample closely followed those from the 829-review matched sample. The correlations between the two samples’ log-OR vectors were 0.998 for the bivariate estimates and 0.996 for the covariate-controlled estimates. Neither covariate-controlled family contained an association with q < 0.05 (Supplementary Table 24).

Visible heritage content was present in 787 of the 829 matched reviews. Among these reviews, 375 also contained heritage-related discourse, corresponding to a conditional concurrence of 47.65% (95% CI [44.18%, 51.14%]). Concurrence was 40.51% among single-sentence reviews (158/390, 95% CI [35.76%, 45.45%]) and 54.66% among multi-sentence reviews (217/397, 95% CI [49.74%, 59.49%]). The corresponding odds ratio was 1.77 (95% CI [1.33, 2.34], Fisher *p* < 0.001). Among multi-sentence reviews containing visible heritage content, 45.34% contained no heritage-related discourse (Table 6 and Supplementary Table 26).

**TABLE 6:** Visible-heritage and heritage-related discourse concurrence by sentence-count group.

Group	Matched N	Visible-heritage N	Both forms of content N	Concurrence	95% CI
All	829	787	375	47.65%	44.18%–51.14%
Single sentence	413	390	158	40.51%	35.76%–45.45%
Multiple sentences	416	397	217	54.66%	49.74%–59.49%

Visible-heritage reviews contain at least one Building space, Cultural symbol, or Environment landscape image. Concurrent T1/T2/T4 indicates heritage-related discourse in the same review. Conditional concurrence uses visible-heritage reviews as the denominator; 95% CIs are Wilson intervals. Among visible-heritage reviews, multi-sentence versus single-sentence concurrence OR = 1.77 (95% CI 1.33–2.34; Fisher *p* < 0.001).

The high-confidence visual sensitivity analysis classified 6,307 of the 6,975 images (90.42%) as high confidence, and 824 of the 829 matched reviews contained at least one high-confidence image. In the category-specific sensitivity models, the association between T3 Architectural/spatial discourse and Building space was OR = 1.64 (95% CI [1.00, 2.69], *p* = 0.052, q = 0.260), whereas the estimate for T4 Interpretation/knowledge discourse and People/activity context was OR = 1.08 (95% CI [0.72, 1.63], *p* = 0.713, q = 0.757). None of the 35 high-confidence associations met q < 0.05 (Supplementary Tables 15, 16).

### 4.3 Heritage-related discourse and textual valence

In the covariate-controlled model of the 812 core matched reviews, heritage-related discourse was positively associated with complete-review textual valence (B = 0.1212, 95% CI [0.0435, 0.1988], *p* = 0.0022; Table 7).

**TABLE 7:** Heritage-related discourse and textual-valence estimates.

Specification	N	Coefficient (95% CI)	P
Heritage-related discourse; complete-review valence	812	B = 0.1212 [0.0435, 0.1988]	0.0022
Same eligible sample; complete-review valence	617	B = 0.1414 [0.0537, 0.2291]	0.0016
Same eligible sample; remaining-text valence	617	B = 0.0432 [−0.0596, 0.1459]	0.4105
Paired coefficient difference (complete - remaining)	617	Delta B = 0.098 [0.049, 0.147]	<0.001

B denotes the focal OLS coefficient estimated with HC3 robust standard errors. The two *N* = 617 outcome models use identical reviews, predictors, and covariates. The paired coefficient-difference row is estimated from within-review complete-minus-remaining valence and therefore formally tests the difference between the two coefficients.

When the three component categories were entered jointly, their coefficients were collectively associated with textual valence [Wald χ^2^(3) = 12.448, *p* = 0.006]. The coefficient estimates were B = 0.071 for T1 Historical-cultural discourse (*p* = 0.124), B = 0.123 for T2 Scholarly-atmosphere discourse (*p* = 0.011), and B = 0.110 for T4 Interpretation/knowledge discourse (*p* = 0.020). None of the three pairwise coefficient contrasts was statistically significant: T1 versus T2, *p* = 0.440; T1 versus T4, *p* = 0.508; and T2 versus T4, *p* = 0.845 (Supplementary Tables 32–34).

Among the 617 reviews eligible for both complete- and remaining-text analyses, the heritage-related-discourse coefficient was B = 0.1414 for complete-review valence (95% CI [0.0537, 0.2291], *p* = 0.0016) and B = 0.0432 for remaining-text valence (95% CI [−0.0596, 0.1459], *p* = 0.4105). The paired coefficient difference was ΔB = 0.098 (HC3 SE = 0.025, 95% CI [0.049, 0.147], *p* < 0.001) (Table 7 and Supplementary Table 36).

T6 Service- and access-related discourse was negatively associated with complete-review textual valence (B = −0.490, 95% CI [−0.603, −0.377], *p* < 0.001). Its interaction with heritage-related discourse was not statistically significant in either the complete-text model (B = 0.169, *p* = 0.115) or the remaining-text model (B = 0.022, *p* = 0.862) (Supplementary Table 35).

Sensitivity analyses yielded a positive heritage-related-discourse coefficient after exclusion of repeated wording (B = 0.129, 95% CI [0.051, 0.207], *p* = 0.001). Under a common text-only specification, the corresponding coefficient was B = 0.072 (95% CI [0.028, 0.116]) in all 2,798 core text reviews and B = 0.147 (95% CI [0.070, 0.223]) in the 812 matched reviews (Supplementary Tables 40, 41).

### 4.4 Platform star ratings

The platform-rating analysis included 2,798 core reviews. Ratings were concentrated at the upper end of the scale: 69 reviews received one star, 22 received two stars, 154 received three stars, 511 received four stars, and 2,042 received five stars. Five-star reviews therefore accounted for 72.98% of the sample (Supplementary Table 43).

The global parallel-lines test rejected the proportional-odds assumption [χ^2^(21) = 145.902, *p* < 0.001; Supplementary Table 37]. Threshold-specific cumulative-logit models were therefore examined at the four native rating cut points.

Heritage-related discourse was not statistically associated with higher ratings at any of the four thresholds. For rating > 1, the estimated OR was 1.67 (95% CI [0.88, 3.19], *p* = 0.117). The corresponding estimates were OR = 1.04 (95% CI [0.63, 1.73], *p* = 0.877) for rating > 2, OR = 1.11 (95% CI [0.79, 1.55], p = 0.544) for rating > 3, and OR = 1.00 (95% CI [0.82, 1.21], *p* = 0.971) for rating > 4 (Table 8). An HC3 OLS sensitivity model treating the 1–5 rating as a numeric outcome yielded B = 0.024 (95% CI [−0.037, 0.085], *p* = 0.436). Additional rating diagnostics are reported in Supplementary Table 37.

**TABLE 8:** Threshold-specific cumulative-logit estimates for platform star ratings (*N* = 2,798).

Cumulative cutoff	B	OR	95% CI for B	P
Rating > 1	0.516	1.674	−0.130 to 1.161	0.117
Rating > 2	0.040	1.041	−0.468 to 0.548	0.877
Rating > 3	0.104	1.110	−0.233 to 0.441	0.544
Rating > 4	−0.004	0.996	−0.202 to 0.194	0.971

Each row reports the heritage-related-discourse coefficient from a cumulative-logit model using the same covariate specification at one of the four native rating cutoffs. The global proportional-odds assumption was rejected [chi-square(21) = 145.902, *p* < 0.001], so these threshold-specific estimates replace a single pooled odds ratio as the principal rating inference. All four confidence intervals include zero. The numeric-scale HC3 OLS sensitivity estimate was B = 0.024 (95% CI −0.037 to 0.085, *p* = 0.436).

### 4.5 Explicit environmental-quality evaluations

Pre-adjudication agreement between the two authors was evaluated for 697 comparable EEQE coding pairs. Observed agreement was 0.9340 (95% bootstrap CI [0.9154, 0.9512]), and Cohen’s κ was 0.8566 (95% bootstrap CI [0.8156, 0.8941]) (Supplementary Table 19).

After adjudication, 430 of the 700 coded reviews were classified as present, 17 as mixed, 252 as absent, and one as unclear. Combining present and mixed yielded 447 reviews with EEQE presence among 699 determinate cases. Of these, 431 were from the three core academies. Direction coding identified 375 positive, 53 negative, 17 mixed-direction, and two neutral-descriptive cases (Supplementary Table 20).

The assessment of reviews not identified by the candidate-screening procedure yielded a design-weighted false-omission rate of 25.01% (95% CI [19.27%, 30.97%]). Estimated screening recall was approximately 38.34%–38.40% (Supplementary Table 21).

The cross-tabulation of EEQE direction and complete-review textual valence showed variation across the two indicators. Among the 53 reviews with negative environmental-quality evaluation, 18 had positive complete-review valence, four had zero valence, and 31 had negative valence. Among the 375 reviews with positive environmental-quality evaluation, 22 had zero complete-review valence and four had negative valence (Supplementary Table 44).

## 5 Discussion

### 5.1 Selective multimodal representation of educational-heritage environments

The findings indicate that educational-heritage environments are selectively represented across visitor-generated modalities. Visitor-uploaded photographs were strongly concentrated on Building space and Cultural symbol content, which together accounted for 72.46% of all images, whereas review text covered a broader range of architectural, historical-cultural, atmospheric, interpretive, environmental, service-related, and on-site-process content (Figures 3, 4 and Tables 3, 4). This pattern is consistent with the environmental-psychology view that experiences of historically significant places emerge from relations among physical settings, visitor knowledge, and psychological and social processes (Ries and Schwan, 2023). Recent multimodal studies likewise show that text and imagery preserve different components of environmental or heritage experience (Schertz et al., 2026; Lin et al., 2026). More generally, multimodal tourism research emphasizes the value of preserving the distinct information carried by each modality when examining how experiences are represented across media (Yu and Cheng, 2025).

The within-review concurrence results further illustrate this complementarity. Heritage-related discourse occurred in 47.65% of reviews containing visible-heritage imagery. Concurrence was higher in multi-sentence than in single-sentence reviews, yet 45.34% of multi-sentence reviews containing visible heritage content still contained no heritage-related discourse (Figure 6, Table 6, and Supplementary Table 26). The 47.65% concurrence therefore captures co-expression between visible heritage content and heritage-related discourse within the same visitor record. Wang (2024) similarly found that visitor-selected social-media imagery emphasizes different environmental features from a more systematic street-view record. The present findings extend this distinction within individual reviews: even when text and photographs originate from the same visit record, the two modalities preserve different but partly overlapping aspects of the heritage environment.

**FIGURE 6:**
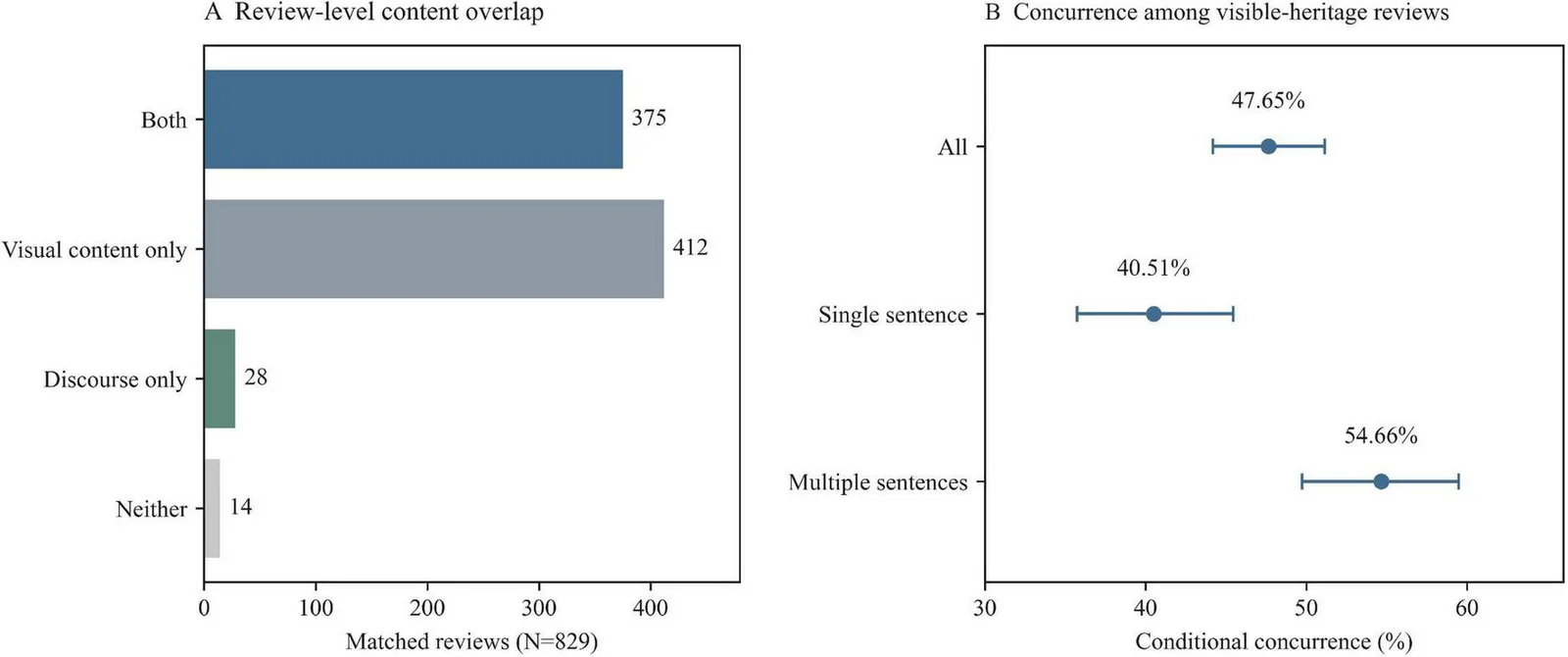
Visible-heritage imagery and heritage-related discourse in matched reviews. **(A)** Shows the four mutually exclusive review groups: both forms of content (375), visual content only (412), discourse only (28), and neither (14), among 829 matched reviews. **(B)** Shows conditional discourse concurrence among reviews with visible-heritage imagery, overall and by sentence count. Points denote proportions and bars denote Wilson 95% confidence intervals. The multi-sentence versus single-sentence odds ratio is 1.77 (95% CI 1.33–2.34; Fisher *p* < 0.001).

The covariate-controlled analysis further shows that text–image correspondence is context dependent. Four topic–image combinations met the FDR criterion in the bivariate analyses, whereas none of the 35 associations met the family-wide FDR criterion after accounting for academy and the amount of textual and visual material available within each review (Figure 5, Table 5, and Supplementary Tables 22, 23). Architectural/spatial discourse and Building space imagery retained the clearest positive alignment in direction, whereas the association between interpretation/knowledge discourse and People/activity context weakened substantially in the high-confidence visual analysis (Supplementary Tables 15, 16). Cross-modal correspondence therefore needs to be interpreted alongside site context and the extent of visitors’ contributions. Visual categories also differ in informational granularity. People/activity imagery, for example, primarily records the visible social setting; identifying guiding, learning, or knowledge communication requires more specific contextual evidence.

Seen from this perspective, the present design complements studies based on direct perceptual measures or external reference systems. Du et al. (2026) compared public expression with a professional design-intention corpus, Zhang et al. (2026) measured visual attention to historical streetscapes using eye tracking, and Fogarasi et al. (2026) examined the psychological meaning of built heritage with a semantic-differential instrument. These approaches capture different aspects of the person–heritage relationship, including external correspondence, visual attention, and place meaning. The present study focuses on how different modalities selectively represent the same educational-heritage environment in post-visit public expression. The findings show that photographs and text preserve different, partly overlapping environmental cues, cultural meanings, and evaluative content. We therefore characterize multimodal educational-heritage UGC in terms of selective multimodal representation, partial cross-modal correspondence, and complementary expression (Figure 1).

### 5.2 Environmental-quality appraisal and layered visitor evaluation

The EEQE results demonstrate the value of retaining the object of evaluation within naturalistic visitor accounts. Within the purposefully constructed 700-review coding sample, 447 of 699 determinate records contained an explicit environmental-quality evaluation, with positive, negative, mixed, and neutral-descriptive directions represented in the coded material (Supplementary Tables 19, 20). Because the sample combined candidate-enriched records with a sample of reviews not identified by screening, these counts characterize the coded sample; estimating EEQE prevalence in the full corpus would require more complete coverage; the performance of the candidate-screening procedure is documented in Supplementary Table 21. Object-specific environmental-quality judgments did not always align with complete-review textual valence: negative EEQE could occur in otherwise positive reviews, while positive EEQE could occur in reviews with zero or negative net valence (Supplementary Table 44). This distinction is consistent with environmental-psychology research that measures environmental appraisal separately from emotional response (Yang et al., 2024), as well as recent UGC studies that distinguish particular environmental qualities within online reviews (Song et al., 2026; Mohamed and Kronenberg, 2025). In educational-heritage settings, EEQE thus retains whether visitors explicitly evaluate scholarly or historical atmosphere, tranquility, artificiality, or other qualities attributed to the setting.

The same-sample comparison of complete and remaining text further locates the association between heritage-related discourse and textual valence. Heritage-related discourse was positively associated with complete-review valence, whereas the association was substantially weaker after T1/T2/T4 sentences were removed from the identical 617 reviews; the formal coefficient difference was ΔB = 0.098 (95% CI [0.049, 0.147], *p* < 0.001) (Table 7 and Supplementary Table 36). This difference indicates that heritage-related content and evaluative language were concentrated within the same textual segments. When visitors discussed historical culture, scholarly atmosphere, or interpretation, they often expressed approval, disappointment, or other evaluations at the same time, whereas this association weakened markedly in the remaining text. Zhou and Liu (2026) likewise found that different cultural-ecosystem-service contents were associated with different emotional patterns, while Ries and Schwan (2024) found that informing participants about a building’s history as a National Socialist clinic led to less positive mood and site evaluations, without improving memory for historically relevant photographs. Together, these findings suggest a contextualized co-expression of heritage content and evaluative language within visitor narratives.

Platform star ratings captured a more compressed level of overall evaluation. Heritage-related discourse showed no statistically reliable association across the four cumulative rating thresholds, and the numeric-rating sensitivity estimate was also close to zero (Table 8 and Supplementary Table 37). Recent tourism-review research has reported both broad alignment between sentiment and ratings (Bigne et al., 2023) and cases of inconsistency (Wang P. et al., 2025). Our analyses examine the association of heritage-related discourse with each evaluative indicator, so these results address a different question from the direct relationship between textual sentiment and star ratings. A platform rating summarizes the visit in a single numerical judgment, while review text retains particular objects of praise and criticism. The concentration of 72.98% of core reviews at five stars further limited differentiation in the rating measure. The results support retaining the content and level of evaluation when interpreting visitor feedback.

Service- and access-related discourse formed another relatively clear evaluative strand. T6 was the only textual topic in which negative sentences outnumbered positive sentences (Figure 3 and Table 3), and it was strongly negatively associated with complete-review textual valence (see Section 4.3). At the same time, the interaction between T6 and heritage-related discourse was weak in both the complete- and remaining-text models (Supplementary Table 35). Overall, service/access concerns and heritage-related appraisal appear to enter visitor narratives largely as parallel forms of evaluative content: practical frictions involving transport, queues, service, or price provide a relatively consistent negative evaluative signal, while visitors may simultaneously evaluate cultural content, scholarly atmosphere, or interpretation. This pattern resembles the distinction between accessibility-related problems and environmental attractiveness made by Mohamed and Kronenberg (2025) and further highlights the multidimensional structure of visitor appraisal in heritage settings.

Taken together, visitor appraisal of educational-heritage environments was distributed across several connected forms of expression. Heritage-related discourse captured the cultural and interpretive content visitors discussed; EEQE retained the specific environmental qualities being evaluated; textual valence summarized evaluative direction in language; service/access discourse reflected practical aspects of the visit; and platform ratings condensed the visit into an overall judgment (Figure 1).

### 5.3 Implications and limitations

The main theoretical implication concerns the differentiation of observational levels in environmental-psychological research using digital traces. As summarized in Figure 1, visitor-uploaded photographs, heritage-related discourse, explicit environmental-quality evaluations, textual valence, and platform ratings capture connected but analytically distinct aspects of post-visit public expression. This organization is consistent with environmental-psychology perspectives that distinguish physical settings, attributed meanings, and evaluative or affective responses within person–place relations (Stedman, 2003; Scannell and Gifford, 2010; Lewicka, 2011). Multimodal UGC therefore provides a useful naturalistic complement to direct psychological measurement when each digital indicator is interpreted according to the level of experience it records.

For heritage management, these distinctions support the use of different indicators for different questions. Visual representation can show which material and symbolic features enter public imagery; interpretation-related discourse can identify visitors’ requests for explanation; EEQE can locate judgments of atmosphere, tranquility, or artificiality; and service/access discourse can reveal practical difficulties. Such feedback can help managers identify issues for closer examination alongside interpretation provision, visit routes, staffing, and environmental conditions. Yao et al. (2026) situate digital communication within organizational performance, and Yao et al. (2024) draw attention to the coordination of tourism-related systems. These perspectives suggest that responses to visitor feedback may require cooperation across communication, site operations, and destination services. The academy coefficients in this study describe site-associated variation; the contribution of particular management practices would require direct assessment of those practices.

Several limitations define the scope of these findings. Ctrip users and their published content are self-selected, and the study covers four Chinese academies on one platform. Platform differences in destination representation have been documented in tourism research (Guo et al., 2021). The 812 core matched reviews also form a selected subset of the 2,798 core text reviews; the sample comparison in Supplementary Table 41 indicates differences in the discourse–valence association. Generalization to other platforms and visitor populations therefore requires comparable external data. Visitor-uploaded photographs are selected public representations, and text and image classification retain measurement error. Limited recall of the EEQE candidate screen prevents estimation of EEQE prevalence in the full corpus. The strong concentration of five-star ratings reduces their discriminatory range. Finally, the post-visit design observes public representation and evaluation at one point in the reporting process. Establishing how environmental exposure, understanding, and affect develop over time requires independent temporal or experimental evidence. Subsequent research will seek comparable multimodal records from additional platforms to examine the consistency of these findings and variation associated with platform context. Combining such records with environmental baselines, direct attention measures, institutional interpretation records, and independently administered psychological measures would extend the assessment of visitor–environment relations.

## 6 Conclusion

This study examined how visitors represent and evaluate educational-heritage environments through multimodal user-generated content from four Chinese academies. The findings reveal a differentiated pattern of post-visit expression. Photographs foregrounded architectural space and cultural symbols, while review text extended into historical meaning, scholarly atmosphere, interpretation, environmental experience, service conditions, and the course of the visit. Text and images overlapped only partly within the same reviews, suggesting that visitors use different media to preserve different facets of their encounters with heritage settings. A similar differentiation emerged in evaluation: heritage-related discourse was closely intertwined with evaluative language in the passages where cultural and interpretive content was discussed, explicit environmental-quality coding captured judgments of atmosphere, tranquility, solemnity, artificiality, and related qualities, and service/access discourse reflected the practical conditions of the visit. Platform ratings, by contrast, condensed these more varied experiences into a broader overall judgment.

Taken together, these findings present visitor experience in educational heritage as a layered form of person–environment expression. Material settings, cultural meanings, environmental qualities, practical experience, and overall evaluation are connected in visitors’ accounts, but they become visible through different forms of digital expression. By bringing photographs, heritage-related discourse, explicit environmental-quality appraisal, textual valence, and platform ratings into the same analytical framework, this study provides a more differentiated way of reading naturally occurring visitor records. Its contribution lies in showing how multimodal UGC can be used to examine visitor–environment relations at scale while retaining the distinct informational roles of different observational layers, thereby extending the methodological possibilities for environmental-psychological research in educational-heritage settings.

## Data Availability

The data supporting the conclusions of this article are available from the corresponding author FL, liangf@wxut.edu.cn, upon reasonable request.
