# Supplementary material for: Selective multimodal representation and visitor appraisal in educational heritage: evidence from Chinese academies
Source: Front Psychol. 2026 Oct 27;17:1926279. doi: 10.3389/fpsyg.2026.1926279 (PMC13747867; doi:10.3389/fpsyg.2026.1926279)
Supplement: Supplementary file 1 [file Table_1.DOCX]

**Supplementary Material**

**Table S1. Corpus coverage by academy**

| **Site** | **Role** | **Valid reviews** | **Valid sentences** | **Valid images** | **Matched reviews** | **Visible-heritage reviews** | **Dominant visual category** |
| --- | --- | --- | --- | --- | --- | --- | --- |
| Yuelu | Core | 1817 | 2780 | 3851 | 478 | 446 | 389 |
| Bailudong | Core | 459 | 765 | 1479 | 163 | 159 | 129 |
| Songyang | Core | 523 | 864 | 1484 | 171 | 165 | 124 |
| Yingtian | Exploratory | 56 | 122 | 161 | 17 | 17 | 13 |
| Total | - | 2855 | 4531 | 6975 | 829 | 787 | 655 |

*Note. The total review corpus contains 2,855 valid reviews. The sentence-level prediction file covers 2,854 reviews and 4,531 valid sentences; this one-review difference does not change the corpus definition.*

**Table S2. Corpus counts and analytical units.**

| **Quantity** | **N** | **Analytical use** |
| --- | --- | --- |
| Valid reviews | 2,855 | Overall review-corpus denominator |
| Valid sentences | 4,531 | Sentence-topic and sentiment denominator |
| Reviews with valid sentences | 2,854 | Sentence-linked review denominator |
| Valid images | 6,975 | Image-level denominator |
| Image-side review identifiers | 835 | Image-projection scope |
| Text–image matched reviews | 829 | Cross-modal association denominator |

**Table S3. Analytical samples and remaining-text eligibility.**

**Panel A. Dataset-specific analytical counts**

| **Quantity** | **Scope/source** | **N** | **Interpretation** |
| --- | --- | --- | --- |
| Valid reviews in the corpus | All four sites | 2,855 | Corpus total; one review has no aggregable sentence |
| Reviews with aggregable sentences and valence | All four sites | 2,854 | Sentence-linked review set |
| Reviews with aggregable sentences and valence | Three core sites | 2,798 | Full core text comparison sample |
| Valid sentences | All four sites | 4,531 | Sentence-level prediction rows |
| T1/T2/T4 sentences removed only from remaining-text outcomes | All four sites | 1,366 | 455 T1 + 314 T2 + 597 T4 |
| Sentences remaining for remaining-text outcomes | All four sites | 3,165 | Non-T1/T2/T4 sentences |
| Review IDs in image-level projection | Image-side projection | 835 | Separate image-side count reported in Table S2 |
| Sentence-linked reviews with an image flag | All four sites | 830 | Text-side raw flag; not equivalent to 835 |
| Sentence-linked reviews with an image flag | Three core sites | 813 | Text-side raw flag |
| Successful text-image matched reviews | All four sites | 829 | Review-level multimodal sample |
| Successful text-image matched reviews | Three core sites | 812 | Main OLS sample |
| Visible-heritage matched reviews | All four sites | 787 | Visible-heritage conditional-occurrence denominator |
| Visible-heritage matched reviews | Three core sites | 770 | Core visible-cue sample |
| Remaining-text eligible reviews | All sentence-linked reviews | 2,076 | At least one non-T1/T2/T4 sentence |
| No remaining sentence | All sentence-linked reviews | 778 | Remaining-text outcomes missing, not zero-filled |
| Remaining-text eligible reviews | Core text reviews | 2,031 | Of 2,798 core text reviews |
| No remaining sentence | Core text reviews | 767 | Of 2,798 core text reviews |
| Remaining-text eligible reviews | All matched reviews | 634 | Of 829 matched reviews |
| No remaining sentence | All matched reviews | 195 | Remaining-text outcomes missing |
| Remaining-text eligible reviews | Core matched reviews | 617 | Remaining-text analysis sample |
| No remaining sentence | Core matched reviews | 195 | Excluded only from residual-outcome models |

**Panel B. Eligibility by academy and T1/T2/T4 presence in the core matched sample**

| **Site** | **T1/T2/T4 presence** | **Original N** | **Remaining-text eligible N** | **No-residual-sentence N** | **Eligible share** |
| --- | --- | --- | --- | --- | --- |
| Bailudong | 0 | 101 | 101 | 0 | 100.00% |
| Bailudong | 1 | 62 | 32 | 30 | 51.61% |
| Songyang | 0 | 103 | 103 | 0 | 100.00% |
| Songyang | 1 | 68 | 35 | 33 | 51.47% |
| Yuelu | 0 | 208 | 208 | 0 | 100.00% |
| Yuelu | 1 | 270 | 138 | 132 | 51.11% |
| Core total | 0 | 412 | 412 | 0 | 100.00% |
| Core total | 1 | 400 | 205 | 195 | 51.25% |
| Core total | All | 812 | 617 | 195 | 75.99% |

*Note. The 835 image-side review identifiers, 830 sentence-linked reviews with an image flag, and 829 successfully matched reviews refer to different sets of records. Review-level multimodal analyses use successful matches containing both valid text and valid images. Reviews without a remaining sentence have missing remaining-text outcomes.*

**Table S4. Shared textual topic framework**

| **Code** | **Content category** | **Observed coverage** |
| --- | --- | --- |
| T1 | Historical-cultural discourse | History, cultural value, and traditional significance |
| T2 | Discourse on scholarly atmosphere | Scholarly atmosphere and cultural-depth discourse |
| T3 | Architectural heritage and spatial form | Buildings, courtyards, halls, and spatial layout |
| T4 | Interpretation- and knowledge-related discourse | Interpretation needs, guide/service evaluation, knowledge references, and self-reported understanding |
| T5 | Environmental landscape and leisure experience | Landscape, trees, scenery, and leisure experience |
| T6 | Service- and access-related discourse | Transport, queues, facilities, tickets, and service content |
| T7 | On-site process and localized experience | Visit process, routes, activities, and other localized content |

*Note. Heritage-related discourse is present when at least one sentence is assigned to T1, T2, or T4. The union represents content coverage. T4 includes interpretation requests, criticism of guiding, and self-reported understanding. Table S8 provides examples, and Table S34 reports component-specific models.*

**Table S5. Model and parameter settings**

| **Module** | **Model** | **Settings** | **Evaluation** |
| --- | --- | --- | --- |
| Review-level topic discovery | BERTopic with KMeans | 1,037 nonempty review documents; k=5–8 comparison; multilingual MiniLM embeddings. Settings for the k=5, 6, and 8 comparison specifications are detailed in Table S7. | Seven categories retained for their content distinctions; review-level comparisons and category mapping in Table S7. |
| Separate sentence diagnostic | KMeans on MiniLM embeddings | 4,217 unique core sentence texts; L2 normalization; k=5–8; n_init=20; max_iter=300; random_state=20260309. | Metrics concern these sentence fits, separately from review discovery (Tables S6 and S7). |
| Sentence-topic projection | Nearest original seven-topic prototype | sentence-transformers/paraphrase-multilingual-MiniLM-L12-v2; arithmetic mean of discovery-document embeddings within each cluster; largest cosine similarity assigns the main topic. Auxiliary mentions use top-two scores ≥0.30. | Two-author stratified validation: 300 sentences; Cohen’s κ=0.687; accuracy against adjudicated labels=0.793. |
| Sentence sentiment | hfl/chinese-roberta-wwm-ext | Review-grouped 490/105/105 sentence split; max length=128; 8 epochs; validation macro-F1 highest at epoch 6; learning rate=2e-5; batches=16/32; weight decay=0.01; random_state=42. | Held-out test: 105 sentences; accuracy=0.8952; macro-F1=0.8858; weighted-F1=0.8939 (Tables S10–S12). |
| Image classification | openai/clip-vit-base-patch32 + logistic regression | 512-dimensional L2-normalized features; StandardScaler; C=4.0; max_iter=5000; class_weight=balanced; random_state=42. | Held-out N=215; accuracy=0.800; macro-F1=0.769. All 6,975 projected labels remain in the primary analyses (Table S15). |
| Repeated-wording sensitivity | Within-corpus text-comparison rules | Same academy, different reviews; Unicode normalization; exact match ≥15 characters or four-gram similarity ≥0.80 with length safeguards. | Flagged sentences are removed only for the sensitivity indicator; no semantic relabeling (Table S40). |

*Note. The two authors independently coded 300 stratified sentences and resolved disagreements by consensus. Inter-coder κ=0.687; top-1 accuracy against adjudicated labels=0.793. This validation and the separate sentence-clustering diagnostic address different aspects of the topic framework.*

**Table S6. Sentence-clustering diagnostics across four resolutions.**

| **k** | **Silhouette (cosine)** | **Calinski- Harabasz** | **Davies- Bouldin** | **Content observed in sentence clusters** | **Diagnostic interpretation** |
| --- | --- | --- | --- | --- | --- |
| k=5 | 0.146 | 326.862 | 2.643 | Broader sentence clusters pool some guide evaluation and general visit/experience expressions. | Highest Calinski–Harabasz and lowest Davies–Bouldin in the diagnostic. |
| k=6 | 0.151 | 297.167 | 2.695 | Some leisure and general visit/evaluation sentences remain pooled; historical and atmospheric content overlaps. | Intermediate resolution with overlapping historical and atmospheric content. |
| k=7 | 0.149 | 271.510 | 2.660 | Seven diagnostic clusters retain semantic overlap and differ from the final seven-category schema. | No common numerical optimum; operational categories are defined by the original review-level solution. |
| k=8 | 0.152 | 249.155 | 2.779 | An interpretable finer subdivision of service/access and visit-process sentences. | Highest cosine silhouette; additional service/access detail remains interpretable. |

*Note. These diagnostics concern 4,217 unique core-site sentence texts, obtained by stripping whitespace and removing exact duplicates from 4,409 projected sentences. They are separate from the original BERTopic discovery on 1,037 review documents. Cosine silhouette uses L2-normalized embeddings. The k=8 solution has the largest silhouette, whereas k=5 has the largest Calinski–Harabasz and smallest Davies–Bouldin values. These criteria do not establish a common optimum. Diagnostic cluster IDs do not define T1–T7; the original discovery clusters have a separate code-defined mapping (Table S7).*

**Table S7. Topic discovery, sentence projection, and resolution diagnostics.**

Topic discovery used 1,037 nonempty review documents. The seven-category framework was retained to distinguish the discourse contents used in the subsequent analyses. Panel E compares the original review-level solutions, and Panel F gives the seven-cluster category mapping. A separate KMeans comparison on 4,217 unique core-site sentences evaluates variation in sentence clustering across four resolutions.

**Panel A. Discovery and sentence-analysis samples**

| **Analysis** | **Units** | **Review scope** | **Construction** |
| --- | --- | --- | --- |
| Balanced discovery sample | 1,038 reviews | 346 per core site | Sample without replacement; seed=20260310 |
| Nonempty discovery documents | 1,037 reviews | Bailudong 346; Songyang 346; Yuelu 345 | One document empty after discovery-specific stopword removal |
| Core-site sentence projection | 4,409 sentences | 2,798 reviews | Input scope for the separate sentence diagnostic |
| Separate sentence diagnostic | 4,217 sentences | 2,738 reviews | Whitespace stripping and exact-text deduplication remove 192 sentence rows |
| Four-site sentence projection | 4,531 sentences | 2,854 reviews | 4,409 core-site plus 122 Yingtian sentences |

*Note. The sampling program restricts eligibility to the three core sites, the topic-discovery flag, and at least three filtered tokens. The eligible pool contains 2,198 reviews (Yuelu 1,463; Bailudong 346; Songyang 389). Sampling without replacement at the smallest site count selects 346 reviews per site (seed=20260310). One Yuelu document becomes empty after discovery-specific stopword removal, giving 1,038 sampled reviews and 1,037 analyzed documents. This exclusion is distinct from the 192 duplicate sentence rows removed for the later diagnostic.*

**Panel B. Separate sentence-clustering metrics and cluster sizes**

| **k** | **N** | **Cosine silhouette** | **Calinski–Harabasz** | **Davies–Bouldin** | **Min–max cluster** |
| --- | --- | --- | --- | --- | --- |
| 5 | 4,217 | 0.146242 | 326.862 | 2.643 | 721–1161 |
| 6 | 4,217 | 0.150812 | 297.167 | 2.695 | 537–837 |
| 7 | 4,217 | 0.148879 | 271.510 | 2.660 | 443–812 |
| 8 | 4,217 | 0.152415 | 249.155 | 2.779 | 309–795 |

*Note. The sentence-level comparison uses sentence-transformers/paraphrase-multilingual-MiniLM-L12-v2, batch size 64, L2-normalized embeddings, KMeans n_init=20, max_iter=300, and seed=20260309. Silhouette is highest at k=8; the Calinski–Harabasz and Davies–Bouldin criteria favor k=5.*

**Panel C. Resolution comparisons in the separate sentence diagnostic**

| **Comparison** | **Full-assignment observation** | **Limited interpretation** |
| --- | --- | --- |
| k=5 vs k=7 | k=5 cluster 3 receives 438/468 from k=7 cluster 3 and 323/443 from k=7 cluster 6. | Some guide and general visit expressions are pooled. |
| k=6 vs k=7 | k=6 cluster 2 receives 424/540 from k=7 cluster 1 and 295/443 from k=7 cluster 6. | Additional resolution partly separates leisure and general visit/evaluation content. |
| k=7 vs k=8 | k=7 cluster 2 splits mainly into k=8 clusters 3 (443/707) and 7 (256/707). | k=8 has an interpretable service/access subdivision. |
| Raw k=7 vs final categories | k=7 cluster 0 contains T3=286, T1=223, T5=124; cluster 5 contains T2=198, T1=178, T3=142. | Raw clusters do not map one-to-one to final T1–T7 labels. |

*Note. Semantic descriptions were based on five sentences of 10–100 characters per diagnostic cluster, giving 130 sentences across 26 clusters. Examples were selected after sorting sentence identifiers, using seeds defined as 20260905 + 100k + cluster ID. These examples describe the content of the sentence clusters; the original review-level comparisons are reported separately in Panel E.*

**Panel D. Discovery settings and sentence-projection rules**

| **Component** | **Method and source scope** |
| --- | --- |
| Discovery text | Filtered tokens were split at the delimiter, 32 discovery-specific stopwords removed, and remaining tokens joined with spaces. |
| Review-level comparison settings | The k=5, 6, and 8 comparison specifications use BERTopic with KMeans and multilingual MiniLM; n_init=20, seed=20260309, token n-grams (1,2), min_df=3, top_n_words=15, calculate_probabilities=False. |
| Dimensionality reduction | The k=5, 6, and 8 comparison specifications use the BERTopic default dimensionality-reduction component. |
| Sentence prototypes | sentence-transformers/paraphrase-multilingual-MiniLM-L12-v2 encodes each original discovery document (batch=32); the arithmetic mean within each original seven-topic cluster forms its prototype. |
| Main and auxiliary assignments | Minimal whitespace, zero-width, and BOM cleaning; batch=32. Largest cosine similarity determines the main category with no rejection threshold. Auxiliary mention flags use the top-two scores ≥0.30. |
| Full sentence application | The category assignment and auxiliary-mention rules were applied to 4,531 sentences from 2,854 reviews. |

*Note. The sentence prototypes were constructed from the original seven review-level discovery clusters. Their mapping to T1–T7 is listed in Panel F. The sentence-level k=5–8 comparison uses a different analytical unit and is reported separately in Panels B and C.*

**Panel E. Original review-level BERTopic discovery outputs**

| **k** | **Original topic sizes** | **Original terms and representative token documents** |
| --- | --- | --- |
| 5 | 240, 216, 212, 186, 183 | Broad historical-cultural content; ticket/access logistics; guide evaluation; scenery/value/experience expressions; architectural-historical description. |
| 6 | 233, 179, 174, 160, 149, 142 | Cluster 0 contains exactly 117 reviews from original k=7 T1 and 116 from T2; historical-cultural and scholarly-atmosphere content is pooled at this coarser resolution. |
| 7 | 172, 168, 165, 147, 134, 132, 119 | The retained operational solution separates T1 and T2; all seven source clusters and their code-defined labels are listed in Panel F. |
| 8 | 171, 155, 136, 128, 127, 121, 102, 97 | 169 of 172 reviews in the retained T7 category divide between k=8 clusters 4 and 6; 131 of 134 T5 reviews divide between clusters 4 and 7. These subdivisions add environmental, guiding, and visit-process detail. |

*Note. The representative documents are tokenized discovery inputs. The discovery sample used seed=20260310. The seven-category solution preserves the distinction between historical-cultural and scholarly-atmosphere content and provides separate categories for interpretation, the environment, services, and visit processes. The numerical diagnostics in Panels B and C evaluate the separate sentence-level comparisons.*

**Panel F. Original seven-cluster mapping used for sentence projection**

| **Original cluster** | **Discovery reviews** | **Projected content category** |
| --- | --- | --- |
| 0 | 172 | T7: On-site process and localized experience |
| 1 | 168 | T1: Historical-cultural discourse |
| 2 | 165 | T3: Architectural heritage and spatial form |
| 3 | 147 | T6: Service- and access-related discourse |
| 4 | 134 | T5: Environmental landscape and leisure experience |
| 5 | 132 | T4: Interpretation- and knowledge-related discourse |
| 6 | 119 | T2: Discourse on scholarly atmosphere |

*Note. This mapping applies only to the original review-discovery clusters. The 4,217-sentence diagnostic has independently fitted cluster identifiers. All 4,531 stored sentence labels and score rules were checked against the projection logic without changing labels or recomputing embeddings.*

**Table S8. Illustrative review excerpts and English translations.**

| **Source / target academy** | **Topic** | **Chinese excerpt** | **English translation** | **Coding context** |
| --- | --- | --- | --- | --- |
| BLD_0263 S001 Bailudong | T1 | 作为历史悠久的书院，应该更好的被管理和被推广 | As an academy with a long history, it should be better managed and promoted. | Historical reference and a management recommendation coexist; topic presence is not verified historical understanding. |
| BLD_0088 S002 Bailudong | T2 | 这里不仅有深厚的历史文化，更有秀丽风光，古往今来，诸多文人学者都在此研学、会友，留下了众多经典佳作 | This place has both a rich history and culture and beautiful scenery. Across the ages, many scholars have studied and met friends here, leaving numerous classic works. | Historical description is expressed alongside appreciation of the setting; the example illustrates the content included in the topic. |
| BLD_0263 S002 Bailudong | T4 | 没有看到讲解，自己在书院里转了转，一知半解 | I did not find any interpretation, so I walked around the academy on my own and understood only bits and pieces. | Unmet interpretation needs and limited self-reported understanding are included in T4. |
| YL_0248 S001 Yuelu | T4 | 讲解小妹妹感觉就是为了完成任务一样，一点都没有服务意识，解说的感觉就是把想让我们知道的读完就好 | The young guide seemed to be just completing a task, with no sense of service; the explanation felt like she only wanted to finish reading what she wanted us to know. | A criticism of guiding service is not a successful learning outcome. |
| YL_0433 S001 Yuelu | T4 | 通过她的讲述我们对书院的历史、传承的精神等有了深一步的了解 | Through her account, we gained a deeper understanding of the academy’s history and the spirit it has passed down. | A contiguous excerpt from a favorable review of the guide. It is self-reported understanding, without an independent learning test. |
| SY_0378 S001 Songyang | T7 | 知道了什么是程门立雪 | I learned what the story of “waiting in the snow at Master Cheng’s gate” means. | Self-reported understanding can occur outside T1/T2/T4, illustrating incomplete semantic coverage of the union. |
| BLD_0234 S002 Bailudong | T1 | 一堆研学的高中生，闹哄哄的，直接在书院学堂里面吃饭，乱七八遭 | A group of high-school students on a study visit were making a racket and eating right inside the academy’s teaching hall; it was a mess. | The full review targets Bailudong and also criticizes access. This excerpt illustrates disturbance and a topic-assignment limitation, not a general activity effect. |
| YL_0395 S001 Yuelu | T6 | 大年初三下雨天，游客超级多，影响了体验感，应该找个淡季好好安静的看历史 | It was raining on the third day of the Lunar New Year and there were so many visitors that it affected the experience. I should come in the off-season to explore the history quietly. | The original rating is five stars. This is a separate illustrative rating case, not one of the machine-valence disagreement counts in Table S44. |

*Note. Chinese excerpts are exact sentences or contiguous sentence portions in the corpus. Review and sentence identifiers preserve source linkage; translations retain the local context. Assigned topics are the projected labels. The examples illustrate topic content and classification limitations. Repeated wording is examined in Table S40.*

**Table S9. Two-author validation of sentence-topic assignments.**

| **Quantity** | **Result** | **Interpretation** |
| --- | --- | --- |
| Validation sample | 300 sentences from 279 reviews | Stratified by academy × assigned topic across 27 non-empty strata |
| Independent coders | Two paper authors | Fixed seven-category codebook; model-assigned topic concealed during independent coding |
| Cohen's kappa | 0.687 | Inter-coder reliability before consensus adjudication |
| Adjudicated top-1 accuracy | 0.793 | Sentence-topic assignment compared with adjudicated human reference labels |
| Measure assessed | Topical content classification | Not a direct measure of psychological understanding, originality, or internalization |

*Note. The 300-sentence validation concerns the reliability of the operational topic-assignment framework. Consensus adjudication resolves coder disagreements for the reference label; the reported kappa refers to the two authors’ independent pre-adjudication coding.*

**Table S10. Review-grouped sentiment training, validation, and test partitions.**

| **Partition** | **Sentences** | **Distinct reviews** | **Negative** | **Neutral** | **Positive** |
| --- | --- | --- | --- | --- | --- |
| Training | 490 | 448 | 118 | 123 | 249 |
| Validation | 105 | 96 | 26 | 26 | 53 |
| Test | 105 | 97 | 26 | 26 | 53 |
| Total | 700 | 641 | 170 | 175 | 355 |

*Note. The 700 reference-labeled sentences were split into 490/105/105 training/validation/test sentences from 448/96/97 reviews. Review IDs do not overlap across partitions. Tables S11, S12 report the held-out evaluation (94/105 correct). The 200-sentence pilot yielded agreement of 89.0% and κ=0.7881 between the two authors; 22 disagreements were adjudicated before labels were finalized.*

**Table S11. Review-grouped sentiment test classification report**

| **Class / summary** | **Precision** | **Recall** | **F1** | **Support** |
| --- | --- | --- | --- | --- |
| Negative | 0.8571 | 0.9231 | 0.8889 | 26 |
| Neutral | 0.9524 | 0.7692 | 0.8511 | 26 |
| Positive | 0.8929 | 0.9434 | 0.9174 | 53 |
| Accuracy | — | — | 0.8952 | 105 |
| Macro average | 0.9008 | 0.8786 | 0.8858 | 105 |
| Weighted average | 0.8988 | 0.8952 | 0.8939 | 105 |

**Table S12. Review-grouped sentiment test confusion matrix**

| **Reference class** | **Predicted negative** | **Predicted neutral** | **Predicted positive** |
| --- | --- | --- | --- |
| Negative | 24 | 0 | 2 |
| Neutral | 2 | 20 | 4 |
| Positive | 2 | 1 | 50 |

**Table S13. Per-category image-classification validation metrics**

| **Class** | **Precision** | **Recall** | **F1 score** | **Support** |
| --- | --- | --- | --- | --- |
| Building space | 0.812 | 0.821 | 0.817 | 84 |
| Cultural symbol | 0.821 | 0.767 | 0.793 | 60 |
| Environment landscape | 0.822 | 0.881 | 0.851 | 42 |
| People/activity context | 0.900 | 0.600 | 0.720 | 15 |
| Service/access cue | 0.579 | 0.786 | 0.667 | 14 |

*Note. One author provided the manually assigned and reviewed image reference labels. Held-out performance is based on 215 images from 106 reviews, with no review shared with the 828-image training set. After validation, the final classifier was fitted to all 1,043 reference images.*

**Table S14. Image-classification validation confusion matrix**

| **True class** | **Pred Building space** | **Pred Cultural symbol** | **Pred Environment landscape** | **Pred People/activity** | **Pred Service/access** |
| --- | --- | --- | --- | --- | --- |
| Building space | 69 | 8 | 6 | 1 | 0 |
| Cultural symbol | 6 | 46 | 2 | 0 | 6 |
| Environment landscape | 4 | 1 | 37 | 0 | 0 |
| People/activity context | 4 | 0 | 0 | 9 | 2 |
| Service/access cue | 2 | 1 | 0 | 0 | 11 |

*Note. Rows denote reference labels and columns denote held-out predictions. Accuracy=0.800; balanced accuracy=0.771; macro-F1=0.769; weighted-F1=0.800; top-2 accuracy=0.949.*

**Table S15. Image validation, confidence groups, and score thresholds.**

**Panel A. Held-out validation and final projection**

| **Quantity** | **Result** |
| --- | --- |
| Integrated image references | 1,043 images from 526 reviews: 818 retained labels + 225 reviewed labels |
| Held-out predictions | 215 images from 106 reviews; 172 correct top-1 predictions |
| Held-out reference linkage | Each of the 215 held-out predictions was linked to its image, review identifier, and human reference label. |
| Review-grouped split | 828 training images / 420 reviews; 215 validation images / 106 reviews; zero review overlap |
| Performance breakdown | Category-specific metrics and the confusion matrix are reported in Tables S13–S14. |
| Accuracy / macro-F1 | 0.8000 / 0.7694 |
| Balanced accuracy / top-2 accuracy | 0.7710 / 0.9488 |
| Splitter | scikit-learn 1.7.2; StratifiedGroupKFold first of five folds; shuffle=True; seed=42 |
| Validation and final fits | The validation classifier used 828 training references; the final classifier used all 1,043 references. |
| Full projection | 6,975 images from 835 reviews; every assigned category equals its stored top-1 prediction |

*Note. The original image split used StratifiedGroupKFold with scikit-learn 1.7.2 (first of five folds, shuffle=True, seed=42), grouped by review. It contains 828 training and 215 held-out images with no review overlap.*

*Note. Image processing converts inputs to RGB, applies the openai/clip-vit-base-patch32 AutoProcessor, and extracts 512-dimensional L2-normalized CLIP features (float32, batch size 32). StandardScaler precedes logistic regression (C=4.0, max_iter=5000, class_weight=balanced, random_state=42, solver=lbfgs; scikit-learn 1.7.2). The validation estimator uses 828 training references; a separate final estimator uses all 1,043 references.*

*Note. One author manually assigned and reviewed the five-category image labels. The final reference set comprises 818 retained labels and 225 eligible labels from a 240-image review set. Fifteen images with mixed or ambiguous content were excluded from that review set. The label definitions concern visible image content; the held-out comparison assesses agreement between the classifier and these reference labels.*

**Panel B. Image-confidence categories**

| **Confidence rule** | **Images** | **Share** |
| --- | --- | --- |
| High: no review flag; top-1 ≥0.80 and margin ≥0.30 | 6,307 | 90.42% |
| Medium: neither high nor review-recommended | 428 | 6.14% |
| Low probability: <0.60 | 240 | 3.44% |
| Low margin: top-1 minus top-2 <0.15 | 148 | 2.12% |
| Recommended review: low probability OR low margin | 240 | 3.44% |

*Note. A review flag is assigned when the highest category score is below 0.60 or the top-1/top-2 margin is below 0.15. The high-confidence rule requires a highest score of at least 0.80, a margin of at least 0.30, and no review flag. All 148 low-margin images also have scores below 0.60. The complete high-confidence rule retains 6,307 images, and 428 intermediate-confidence images have scores from 0.60 to below 0.80. The 0.80 cutoff provides a selective sensitivity subset with 90.42% corpus coverage; all 6,975 labels remain in the primary analyses. These scores express the classifier’s relative confidence. Classification accuracy is evaluated separately on held-out reference images in Tables S13–S14.*

**Panel C. Descriptive image retention at alternative score thresholds**

| **Minimum score** | **Images retained** | **Share** | **Image-side reviews** | **Review–label presences** | **Presences lost** |
| --- | --- | --- | --- | --- | --- |
| 0.00 | 6,975 | 100.00% | 835 | 1,959 | 0 |
| 0.60 | 6,735 | 96.56% | 834 | 1,918 | 41 |
| 0.70 | 6,534 | 93.68% | 832 | 1,877 | 82 |
| 0.80 | 6,307 | 90.42% | 830 | 1,837 | 122 |
| 0.90 | 5,930 | 85.02% | 823 | 1,768 | 191 |

*Note. A review–label presence pairs one image-side review identifier with at least one image of a category. Its scope differs from the final 829-review matched matrix. Class-frequency ranks do not change across the displayed thresholds. These checks describe retention and composition; they do not remove observations from the primary analyses. Table S16 reports the category-specific high-confidence sensitivity for the four combinations shown in main-text Table 5.*

**Table S16. High-confidence visual sensitivity for four topic–image combinations.**

| **Text × visual pair** | **Full-data adjusted OR** | **Full-data BH q** | **High-confidence known N** | **High-confidence adjusted OR (95% CI)** | **p** | **BH q** |
| --- | --- | --- | --- | --- | --- | --- |
| T3 × Building space | 1.55 | 0.247 | 757 | 1.64 (1.00–2.69) | 0.052 | 0.260 |
| T4 × Building space | 0.84 | 0.608 | 757 | 0.67 (0.40–1.12) | 0.124 | 0.482 |
| T4 × Environment landscape | 0.67 | 0.247 | 629 | 0.58 (0.36–0.94) | 0.028 | 0.223 |
| T4 × People/activity context | 1.50 | 0.229 | 580 | 1.08 (0.72–1.63) | 0.713 | 0.757 |

*Note. A visual category is present when at least one high-confidence image belongs to it. Absence requires all linked images to be high confidence and none to belong to the category; otherwise the category is unresolved. Models adjust for academy, log(1+valid sentence count), and log(1+high-confidence image count), with Huber–White standard errors. All 35 high-confidence results are provided in the data package; none meets BH q<0.05.*

**Table S17. Review-level observational indicator definitions**

| **Indicator** | **Definition** | **Unit** | **Interpretation** |
| --- | --- | --- | --- |
| Visible heritage imagery | At least one image of a building, cultural symbol, or landscape | Review | Defines the conditional-occurrence sample |
| People/activity image presence | At least one image classified as people/activity context | Review | Photographed social context; no direct learning measure |
| T1 presence | At least one sentence assigned to T1 | Review | Historical-cultural topical content |
| T2 presence | At least one sentence assigned to T2 | Review | Atmosphere-related topical content |
| T4 presence | At least one sentence assigned to T4 | Review | Interpretation/knowledge content, including needs and criticism |
| Heritage-related discourse | At least one sentence assigned to T1, T2, or T4 | Review | Topic-assignment union, without further semantic screening |
| Repetition-screened expression | T1/T2/T4 union after removal of flagged repeated sentences | Review | Sensitivity to repeated wording |
| T1/T4 union | At least one T1 or T4 sentence | Review | Alternative content coverage excluding T2 |
| T1/T2/T3/T4 union | At least one sentence from any of these four topics | Review | Alternative coverage adding architectural/spatial content |
| Full-review text valence | (Positive sentences − negative sentences)/valid sentences | Review | Model-estimated textual valence, from −1 to 1 |
| T6 presence | At least one sentence assigned to T6 | Review | Service/access-related topical content; covariate |
| Remaining-text eligibility | At least one sentence outside T1/T2/T4 | Review | No remaining sentence means a missing outcome |
| Remaining-text valence | (Remaining positive − remaining negative sentences)/remaining sentences | Review | Outcome after T1/T2/T4 sentence removal |
| Remaining-text probability valence | Mean P(positive) − P(negative) over remaining sentences | Review | Alternative remaining-text outcome |
| Remaining-text positive share | Remaining positive sentences/remaining sentences | Review | Alternative remaining-text outcome |

*Note. Topic and image presences are aggregated within review IDs. Heritage-related discourse is the T1/T2/T4 union. The indicators describe linked public expressions with distinct measurement targets.*

**Table S18. EEQE candidate-screening anchors.**

The initial candidate-screening rules covered five families of environmental-quality expression. The patterns below illustrate the content used to identify potentially relevant reviews and the contextual checks needed during human coding.

| **Rule ID** | **EEQE dimension** | **Documented pattern** | **Known false-positive risk** |
| --- | --- | --- | --- |
| EEQE_HIST_01 | historical_academic_atmosphere | 书香气\|书香\|文化气息\|文化氛围\|历史感\|历史氛围\|学术氛围\|古雅\|古朴\|历史厚重感\|历史厚度 | encyclopedic history or generic cultural praise without environment attribution |
| EEQE_TRANQ_01 | tranquility_serenity | 安静\|清幽\|幽静\|静谧\|清雅\|远离喧闹\|远离喧嚣 | other attractions or service contexts |
| EEQE_REST_01 | explicit_restorative_like_expression | 让人放松\|心静下来\|静下心\|治愈\|舒缓\|远离喧嚣.(轻松\|放松)\|感觉轻松 | activity advice or other-attraction experience; does not prove restoration |
| EEQE_SOLEM_01 | solemnity_depth | 庄重\|肃穆\|厚重\|神圣\|敬意\|沉浸.(历史厚度\|历史\|文化) | generic historical content or respect for a person/culture without an environment-attributed quality |
| EEQE_DIST_01 | disturbance_artificiality | 拥挤\|嘈杂\|商业化\|空洞\|粗陋\|破败\|过度新建\|仿古感强\|缺少历史感\|没有历史感\|人太多\|游客太多\|建筑.太新 | negated disturbance or an ineligible referent |

*Note. These patterns document the initial candidate-screening rules. A match identified text for closer examination; the final EEQE decision required an explicit quality attributed to the target academy or its immediate setting. The coding sample comprised 419 core candidate reviews, 240 core reviews not identified by the screen, and 41 Yingtian reviews. Two authors independently assigned the final labels using the EEQE codebook (Table S19). Screening performance is reported in Table S21.*

**Table S19. EEQE coding design and two-author agreement.**

Explicit environmental-quality expressions (EEQE) attribute a quality to the target academy's social or physical setting. Coding covered historical or scholarly atmosphere, tranquility, explicitly restorative-like wording, solemnity or depth, disturbance or artificiality, and other attributed qualities. Generic praise, historical facts, and object lists alone were insufficient. Target-academy identity was supplied to resolve references such as 'here' or 'the academy', while clearly off-target places or services were distinguished. Two authors independently coded all 700 reviews using the same fixed codebook in separately ordered records. Disagreements were resolved through adjudication. The agreement statistics in this table therefore represent human inter-coder reliability for the content-coding procedure..

| **Design component** | **Description** |
| --- | --- |
| Sample | 700 reviews: 419 core screen-positive candidates, 240 core screen-negative reviews, and 41 Yingtian reviews |
| Coding sources | Two paper authors independently coded all 700 reviews using the fixed EEQE codebook in separately ordered records. |
| Target context | Target academy supplied to identify the object of environmental-quality expression |
| Binary presence | Present or mixed = 1; absent = 0; unclear = missing |
| Pre-adjudication comparison | 697 complete author-coded pairs; three pairs with missing values. |
| Bootstrap | 10,000 review resamples stratified by academy × selection reason; seed=2026090403 |
| Adjudication | Coder disagreements and borderline fields were resolved through structured author adjudication; fields on which the authors already agreed were retained. |
| Final coding | 700 adjudicated records, including one unclear presence label; one composite inconsistency resolved through consistency review. |

**Panel A. Overall binary environmental-quality presence agreement before adjudication**

| **Metric** | **Estimate** | **95% bootstrap CI** |
| --- | --- | --- |
| Observed agreement | 0.9340 | [0.9154, 0.9512] |
| Cohen’s kappa | 0.8566 | [0.8156, 0.8941] |
| Nominal Krippendorff alpha | 0.8566 | [0.8155, 0.8941] |
| Gwet AC1 | 0.8778 | [0.8426, 0.9103] |
| Positive specific agreement | 0.9485 | [0.9338, 0.9624] |
| Negative specific agreement | 0.9080 | [0.8808, 0.9328] |

**Panel B. Selected domains with more limited agreement**

| **Domain** | **Kappa** | **Additional result** | **Scope of interpretation** |
| --- | --- | --- | --- |
| Yingtian binary presence | 0.5505 | n=41; agreement=0.7805 | Small exploratory-site sample |
| Six-category referent scope | 0.6178 | Eligible/off-target binary κ=0.7958 | Finer target distinctions have lower agreement |
| Multiple referents | 0.5288 | Review-level multiple-referent flag | Limited fine-grained agreement |
| Solemnity/depth | 0.5744 | Positive agreement=0.6071 | Does not inherit overall binary agreement |
| Explicitly restorative-like language | 0.7363 | Positive agreement=0.7429; sparse | Expressed content rather than measured restoration |

*Note. Binary presence maps present or mixed expression to one, absent to zero, and unclear to missing. Human inter-coder agreement was computed before adjudication on 697 complete author-coded pairs; three pairs had missing values. Bootstrap intervals use 10,000 review resamples stratified by academy and selection reason (seed=2026090403). Overall binary agreement does not imply equal agreement for every referent distinction or quality dimension. The adjudicated labels retain explicit evidence spans and the original object-specific coding boundaries.*

**Table S20. Adjudicated EEQE labels and related textual indicators.**

**Panel A. Final adjudicated labels in the 700-review sample**

| **Adjudicated presence classification** | **Reviews** |
| --- | --- |
| Present, with one evaluative/descriptive direction | 430 |
| Mixed positive and negative directions | 17 |
| Absent | 252 |
| Unclear | 1 |
| Presence-positive total | 447 |
| Presence-positive core-site reviews | 431 |

*Note. The 447 presence-positive records include 375 positive-direction, 53 negative-direction, 17 mixed-direction, and two neutral descriptive expressions; presence is therefore distinct from positive direction. Of these records, 431 are from the three core academies: Yuelu 233, Bailudong 91, and Songyang 107. Counts describe the 700-review coded design and are not full-corpus prevalence estimates. One presence label remains unclear. Auxiliary authenticity and expectation codes are retained descriptively but are too sparse for strong inference.*

**Panel B. Cross-classification with adjacent indicators; n=699 determinate reviews**

| **Comparator presence** | **Quality present / comparator absent** | **Quality absent / comparator present** | **Phi** |
| --- | --- | --- | --- |
| Generic praise | 362 | 55 | −0.034 |
| T2 | 370 | 11 | 0.186 |
| T6 | 360 | 42 | 0.035 |
| Positive full-review machine valence | 65 | 159 | 0.257 |
| Formulaic wording | 289 | 59 | 0.124 |

*Note. Off-diagonal counts show that object-specific environmental-quality coding and adjacent textual indicators classify some reviews differently. They do not establish psychometric discriminant validity. Of 447 EEQE-positive records, 158 (35.3%) have a formulaic-wording flag and 289 do not. Table S44 reports EEQE direction alongside the sign of complete-review model-estimated text valence for all 699 determinate records.*

**Table S21. EEQE candidate-screening coverage.**

| **Screening-performance quantity** | **Estimate** | **95% survey-bootstrap CI** |
| --- | --- | --- |
| Weighted false negatives | 583.83 | [449.67, 722.80] |
| Weighted true negatives | 1750.17 | [1611.20, 1884.33] |
| Screen-negative FOR | 25.01% | [19.27%, 30.97%] |
| Negative predictive value | 74.99% | [69.03%, 80.73%] |
| Candidate true positives; lower bound | 363.00 | [363.00, 363.00] |
| Candidate true positives; upper bound | 364.00 | [364.00, 364.00] |
| Recall; TP lower bound | 38.34% | [33.43%, 44.67%] |
| Recall; TP upper bound | 38.40% | [33.49%, 44.74%] |

*Note. Screening performance is assessed in the core corpus. A stratified survey-weighted bootstrap uses 10,000 resamples (seed=2026090404). One unclear candidate gives 363–364 true positives. The false-omission rate is 25.01% (95% CI 19.27%–30.97%) and estimated recall is 38.34%–38.40%. EEQE results describe the coded sample; uncoded records are not assigned absence.*

**Table S22. Bivariate topic–image association matrix (N=829).**

| **Topic** | **Visual** | **Both n** | **Topic N** | **Visual N** | **Lift** | **Phi** | **FDR q** |
| --- | --- | --- | --- | --- | --- | --- | --- |
| T1 | Building space | 148 | 187 | 630 | 1.0414 | 0.0398 | 0.5246 |
| T1 | Cultural symbol | 120 | 187 | 581 | 0.9156 | -0.0697 | 0.1806 |
| T1 | Environment landscape | 90 | 187 | 341 | 1.1700 | 0.0767 | 0.1421 |
| T1 | People/activity context | 53 | 187 | 253 | 0.9287 | -0.0255 | 0.7054 |
| T1 | Service/access cue | 36 | 187 | 143 | 1.1160 | 0.0286 | 0.6717 |
| T2 | Building space | 73 | 91 | 630 | 1.0556 | 0.0347 | 0.5786 |
| T2 | Cultural symbol | 61 | 91 | 581 | 0.9565 | -0.0234 | 0.7054 |
| T2 | Environment landscape | 36 | 91 | 341 | 0.9617 | -0.0112 | 0.8960 |
| T2 | People/activity context | 22 | 91 | 253 | 0.7922 | -0.0484 | 0.4565 |
| T2 | Service/access cue | 16 | 91 | 143 | 1.0193 | 0.0031 | 0.9100 |
| T3 | Building space | 321 | 378 | 630 | 1.1174 | 0.1913 | <0.001 |
| T3 | Cultural symbol | 281 | 378 | 581 | 1.0607 | 0.0851 | 0.0870 |
| T3 | Environment landscape | 160 | 378 | 341 | 1.0290 | 0.0222 | 0.7054 |
| T3 | People/activity context | 109 | 378 | 253 | 0.9449 | -0.0335 | 0.5786 |
| T3 | Service/access cue | 63 | 378 | 143 | 0.9662 | -0.0141 | 0.8600 |
| T4 | Building space | 114 | 178 | 630 | 0.8428 | -0.1463 | <0.001 |
| T4 | Cultural symbol | 117 | 178 | 581 | 0.9379 | -0.0497 | 0.4565 |
| T4 | Environment landscape | 47 | 178 | 341 | 0.6419 | -0.1565 | <0.001 |
| T4 | People/activity context | 72 | 178 | 253 | 1.3254 | 0.1128 | 0.0151 |
| T4 | Service/access cue | 21 | 178 | 143 | 0.6839 | -0.0755 | 0.1446 |
| T5 | Building space | 112 | 140 | 630 | 1.0527 | 0.0423 | 0.4565 |
| T5 | Cultural symbol | 92 | 140 | 581 | 0.9376 | -0.0430 | 0.4565 |
| T5 | Environment landscape | 65 | 140 | 341 | 1.1287 | 0.0485 | 0.4565 |
| T5 | People/activity context | 41 | 140 | 253 | 0.9596 | -0.0121 | 0.8907 |
| T5 | Service/access cue | 30 | 140 | 143 | 1.2423 | 0.0499 | 0.4565 |
| T6 | Building space | 138 | 183 | 630 | 0.9923 | -0.0073 | 0.8960 |
| T6 | Cultural symbol | 131 | 183 | 581 | 1.0214 | 0.0174 | 0.8103 |
| T6 | Environment landscape | 77 | 183 | 341 | 1.0229 | 0.0102 | 0.8960 |
| T6 | People/activity context | 60 | 183 | 253 | 1.0743 | 0.0262 | 0.6817 |
| T6 | Service/access cue | 44 | 183 | 143 | 1.3939 | 0.0957 | 0.0537 |
| T7 | Building space | 117 | 162 | 630 | 0.9504 | -0.0435 | 0.4565 |
| T7 | Cultural symbol | 114 | 162 | 581 | 1.0041 | 0.0031 | 1.0000 |
| T7 | Environment landscape | 76 | 162 | 341 | 1.1405 | 0.0579 | 0.3816 |
| T7 | People/activity context | 56 | 162 | 253 | 1.1327 | 0.0433 | 0.4565 |
| T7 | Service/access cue | 32 | 162 | 143 | 1.1451 | 0.0327 | 0.5786 |

**Table S23. Adjusted topic–image association matrix (N=829).**

| **Topic** | **Visual category** | **Adjusted OR** | **95% CI** | **Robust p** | **BH q** |
| --- | --- | --- | --- | --- | --- |
| T1 | Building space | 1.014 | 0.631–1.630 | 0.9538 | 0.9538 |
| T1 | Cultural symbol | 0.580 | 0.388–0.868 | 0.0080 | 0.1212 |
| T1 | Environment landscape | 1.408 | 0.969–2.046 | 0.0724 | 0.2815 |
| T1 | People/activity context | 0.843 | 0.565–1.259 | 0.4048 | 0.6078 |
| T1 | Service/access cue | 1.140 | 0.735–1.767 | 0.5588 | 0.6985 |
| T2 | Building space | 1.521 | 0.801–2.887 | 0.1996 | 0.4657 |
| T2 | Cultural symbol | 1.116 | 0.663–1.878 | 0.6805 | 0.7217 |
| T2 | Environment landscape | 0.974 | 0.587–1.614 | 0.9181 | 0.9451 |
| T2 | People/activity context | 0.753 | 0.432–1.313 | 0.3171 | 0.5551 |
| T2 | Service/access cue | 1.162 | 0.629–2.148 | 0.6320 | 0.7217 |
| T3 | Building space | 1.547 | 1.015–2.357 | 0.0423 | 0.2467 |
| T3 | Cultural symbol | 1.096 | 0.762–1.577 | 0.6196 | 0.7217 |
| T3 | Environment landscape | 0.659 | 0.468–0.928 | 0.0171 | 0.1497 |
| T3 | People/activity context | 0.796 | 0.566–1.120 | 0.1899 | 0.4657 |
| T3 | Service/access cue | 0.686 | 0.447–1.052 | 0.0841 | 0.2943 |
| T4 | Building space | 0.838 | 0.542–1.297 | 0.4285 | 0.6078 |
| T4 | Cultural symbol | 1.386 | 0.909–2.114 | 0.1297 | 0.3783 |
| T4 | Environment landscape | 0.667 | 0.440–1.011 | 0.0565 | 0.2473 |
| T4 | People/activity context | 1.496 | 1.034–2.166 | 0.0328 | 0.2294 |
| T4 | Service/access cue | 0.814 | 0.486–1.363 | 0.4342 | 0.6078 |
| T5 | Building space | 1.176 | 0.693–1.997 | 0.5486 | 0.6985 |
| T5 | Cultural symbol | 0.724 | 0.468–1.119 | 0.1459 | 0.3928 |
| T5 | Environment landscape | 1.284 | 0.845–1.949 | 0.2416 | 0.4869 |
| T5 | People/activity context | 0.818 | 0.527–1.268 | 0.3682 | 0.6078 |
| T5 | Service/access cue | 1.473 | 0.930–2.334 | 0.0989 | 0.3146 |
| T6 | Building space | 0.760 | 0.477–1.213 | 0.2504 | 0.4869 |
| T6 | Cultural symbol | 1.180 | 0.781–1.783 | 0.4310 | 0.6078 |
| T6 | Environment landscape | 1.091 | 0.737–1.613 | 0.6642 | 0.7217 |
| T6 | People/activity context | 1.220 | 0.826–1.800 | 0.3172 | 0.5551 |
| T6 | Service/access cue | 1.918 | 1.233–2.986 | 0.0039 | 0.1212 |
| T7 | Building space | 0.524 | 0.320–0.859 | 0.0104 | 0.1212 |
| T7 | Cultural symbol | 1.101 | 0.700–1.733 | 0.6769 | 0.7217 |
| T7 | Environment landscape | 1.302 | 0.854–1.985 | 0.2195 | 0.4803 |
| T7 | People/activity context | 1.499 | 0.996–2.255 | 0.0522 | 0.2473 |
| T7 | Service/access cue | 1.191 | 0.738–1.921 | 0.4736 | 0.6375 |

*Note. For each pair, visual-category presence is modeled using topic presence, academy, log(1+valid sentence count), and log(1+valid image count), with Huber–White sandwich standard errors. Benjamini–Hochberg correction is applied to all 35 p values. No adjusted association meets q<0.05.*

**Table S24. Comparison of the full and core matched association estimates.**

| **Comparison** | **829 matched reviews** | **812 core matched reviews** | **Cross-sample result** |
| --- | --- | --- | --- |
| Unadjusted 35-test family | 4/35 q<0.05 | 5/35 q<0.05 | Correlation of 35 log ORs=0.9976; max \|Δ log OR\|=0.050 |
| Uniformly adjusted 35-test family | 0/35 q<0.05 | 0/35 q<0.05 | Correlation of 35 log ORs=0.9963; max \|Δ log OR\|=0.100 |
| Sampling relation | Includes 17 Yingtian reviews | Excludes the same 17 Yingtian reviews | Nested sensitivity, not independent replication |

*Note. Adjustment is identical to Table S23: academy, log(1+valid sentence count), and log(1+valid image count), with Huber–White robust standard errors and one BH correction across each 35-test family.*

**Panel C. Joint visual-category model; core N=812**

| **Visual category** | **B** | **HC1 SE** | **95% CI(B)** | **OR** | **p** | **BH q** |
| --- | --- | --- | --- | --- | --- | --- |
| Building | 0.107 | 0.202 | [-0.289, 0.504] | 1.113 | 0.595 | 0.849 |
| Culture | -0.154 | 0.182 | [-0.511, 0.204] | 0.857 | 0.399 | 0.849 |
| Landscape | 0.010 | 0.174 | [-0.330, 0.351] | 1.010 | 0.953 | 0.953 |
| People/activity | -0.071 | 0.172 | [-0.408, 0.266] | 0.931 | 0.680 | 0.849 |
| Service/access | -0.139 | 0.208 | [-0.546, 0.269] | 0.871 | 0.505 | 0.849 |

*Note. This secondary model enters the five visual-presence indicators jointly in a logistic model for T1/T2/T4 presence. It is retained for transparency but is not the principal cross-modal specification. The principal cross-modal inference uses the uniformly adjusted 35-cell family in Table S23, for which no association reaches q<0.05.*

**Table S25. Bivariate topic–image associations in the full and core matched samples.**

**Panel A. All matched reviews, N=829**

| **Topic** | **Visual category** | **Both n** | **OR** | **Lift** | **Phi** | **BH q** |
| --- | --- | --- | --- | --- | --- | --- |
| T1 | Building | 148 | 1.251 | 1.041 | 0.040 | 0.525 |
| T1 | Culture | 120 | 0.702 | 0.916 | -0.070 | 0.181 |
| T1 | Landscape | 90 | 1.445 | 1.170 | 0.077 | 0.142 |
| T1 | People/activity | 53 | 0.878 | 0.929 | -0.026 | 0.705 |
| T1 | Service/access | 36 | 1.200 | 1.116 | 0.029 | 0.672 |
| T2 | Building | 73 | 1.293 | 1.056 | 0.035 | 0.579 |
| T2 | Culture | 61 | 0.846 | 0.956 | -0.023 | 0.705 |
| T2 | Landscape | 36 | 0.933 | 0.962 | -0.011 | 0.896 |
| T2 | People/activity | 22 | 0.710 | 0.792 | -0.048 | 0.456 |
| T2 | Service/access | 16 | 1.048 | 1.019 | 0.003 | 0.910 |
| T3 | Building | 321 | 2.574 | 1.117 | 0.191 | <0.001 |
| T3 | Culture | 281 | 1.456 | 1.061 | 0.085 | 0.087 |
| T3 | Landscape | 160 | 1.095 | 1.029 | 0.022 | 0.705 |
| T3 | People/activity | 109 | 0.865 | 0.945 | -0.033 | 0.579 |
| T3 | Service/access | 63 | 0.929 | 0.966 | -0.014 | 0.860 |
| T4 | Building | 114 | 0.466 | 0.843 | -0.146 | <0.001 |
| T4 | Culture | 117 | 0.771 | 0.938 | -0.050 | 0.456 |
| T4 | Landscape | 47 | 0.438 | 0.642 | -0.157 | <0.001 |
| T4 | People/activity | 72 | 1.765 | 1.325 | 0.113 | 0.015 |
| T4 | Service/access | 21 | 0.590 | 0.684 | -0.075 | 0.145 |
| T5 | Building | 112 | 1.306 | 1.053 | 0.042 | 0.456 |
| T5 | Culture | 92 | 0.781 | 0.938 | -0.043 | 0.456 |
| T5 | Landscape | 65 | 1.297 | 1.129 | 0.049 | 0.456 |
| T5 | People/activity | 41 | 0.937 | 0.960 | -0.012 | 0.891 |
| T5 | Service/access | 30 | 1.402 | 1.242 | 0.050 | 0.456 |
| T6 | Building | 138 | 0.955 | 0.992 | -0.007 | 0.896 |
| T6 | Culture | 131 | 1.093 | 1.021 | 0.017 | 0.810 |
| T6 | Landscape | 77 | 1.052 | 1.023 | 0.010 | 0.896 |
| T6 | People/activity | 60 | 1.148 | 1.074 | 0.026 | 0.682 |
| T6 | Service/access | 44 | 1.755 | 1.394 | 0.096 | 0.054 |
| T7 | Building | 117 | 0.777 | 0.950 | -0.044 | 0.456 |
| T7 | Culture | 114 | 1.013 | 1.004 | 0.003 | 1.000 |
| T7 | Landscape | 76 | 1.341 | 1.141 | 0.058 | 0.382 |
| T7 | People/activity | 56 | 1.264 | 1.133 | 0.043 | 0.456 |
| T7 | Service/access | 32 | 1.243 | 1.145 | 0.033 | 0.579 |

**Panel B. Core matched reviews, N=812**

| **Topic** | **Visual category** | **Both n** | **OR** | **Lift** | **Phi** | **BH q** |
| --- | --- | --- | --- | --- | --- | --- |
| T1 | Building | 146 | 1.273 | 1.045 | 0.043 | 0.473 |
| T1 | Culture | 120 | 0.731 | 0.926 | -0.062 | 0.323 |
| T1 | Landscape | 89 | 1.416 | 1.159 | 0.073 | 0.182 |
| T1 | People/activity | 53 | 0.861 | 0.919 | -0.029 | 0.621 |
| T1 | Service/access | 36 | 1.252 | 1.145 | 0.036 | 0.555 |
| T2 | Building | 72 | 1.314 | 1.060 | 0.037 | 0.605 |
| T2 | Culture | 60 | 0.831 | 0.951 | -0.026 | 0.621 |
| T2 | Landscape | 36 | 0.936 | 0.964 | -0.011 | 0.898 |
| T2 | People/activity | 22 | 0.697 | 0.785 | -0.051 | 0.473 |
| T2 | Service/access | 15 | 0.997 | 0.981 | -0.003 | 1.000 |
| T3 | Building | 309 | 2.519 | 1.118 | 0.188 | <0.001 |
| T3 | Culture | 274 | 1.521 | 1.068 | 0.095 | 0.050 |
| T3 | Landscape | 157 | 1.110 | 1.034 | 0.026 | 0.621 |
| T3 | People/activity | 109 | 0.890 | 0.956 | -0.027 | 0.621 |
| T3 | Service/access | 61 | 0.960 | 0.981 | -0.008 | 0.903 |
| T4 | Building | 114 | 0.482 | 0.848 | -0.141 | 0.001 |
| T4 | Culture | 117 | 0.771 | 0.938 | -0.050 | 0.473 |
| T4 | Landscape | 47 | 0.428 | 0.636 | -0.162 | <0.001 |
| T4 | People/activity | 72 | 1.701 | 1.298 | 0.106 | 0.029 |
| T4 | Service/access | 21 | 0.601 | 0.694 | -0.073 | 0.182 |
| T5 | Building | 111 | 1.335 | 1.058 | 0.046 | 0.473 |
| T5 | Culture | 92 | 0.801 | 0.945 | -0.039 | 0.555 |
| T5 | Landscape | 65 | 1.295 | 1.127 | 0.049 | 0.473 |
| T5 | People/activity | 41 | 0.915 | 0.947 | -0.016 | 0.830 |
| T5 | Service/access | 29 | 1.376 | 1.228 | 0.047 | 0.473 |
| T6 | Building | 132 | 0.934 | 0.988 | -0.011 | 0.882 |
| T6 | Culture | 126 | 1.066 | 1.016 | 0.013 | 0.882 |
| T6 | Landscape | 76 | 1.079 | 1.035 | 0.015 | 0.830 |
| T6 | People/activity | 60 | 1.177 | 1.088 | 0.031 | 0.621 |
| T6 | Service/access | 42 | 1.754 | 1.396 | 0.095 | 0.053 |
| T7 | Building | 113 | 0.770 | 0.947 | -0.045 | 0.473 |
| T7 | Culture | 111 | 1.006 | 1.003 | 0.002 | 1.000 |
| T7 | Landscape | 75 | 1.352 | 1.144 | 0.060 | 0.368 |
| T7 | People/activity | 56 | 1.277 | 1.138 | 0.045 | 0.473 |
| T7 | Service/access | 30 | 1.196 | 1.117 | 0.026 | 0.621 |

*Note. Panels A and B retain the complete unadjusted 35-test matrices for all 829 matched reviews and the nested 812-review core subset. The 812 reviews equal the 829 sample minus 17 Yingtian records and are not an independent replication. The unadjusted effect spectra are nearly identical (correlation of 35 log ORs=0.9976). Under the common adjusted specification used in Table S23, the 829 and 812 effect spectra are likewise highly similar (correlation=0.9963), and both adjusted families yield 0/35 BH-FDR q<0.05. Table S24 summarizes the nested-sample comparison. Odds ratios use the same Haldane–Anscombe correction as main-text Table 5.*

**Table S26. Visible-heritage imagery and discourse concurrence by sentence-count group.**

| **Group** | **N** | **Both n** | **Visual only** | **Discourse only** | **Neither** | **Visible N** | **Rate (%)** | **95% CI** |
| --- | --- | --- | --- | --- | --- | --- | --- | --- |
| Single-sentence reviews | 413 | 158 | 232 | 12 | 11 | 390 | 40.51% | 35.76%–45.45% |
| Multi-sentence reviews | 416 | 217 | 180 | 16 | 3 | 397 | 54.66% | 49.74%–59.49% |
| All matched reviews | 829 | 375 | 412 | 28 | 14 | 787 | 47.65% | 44.18%–51.14% |

*Note. Within reviews already containing visible-heritage image content, multi-sentence reviews were more likely than single-sentence reviews to contain T1/T2/T4 discourse (OR=1.77, 95% CI 1.33–2.34, Fisher p<0.001). Multi-sentence reviews averaged 2.18 review-level topic presences and 77.88% contained more than one topic presence; single-sentence reviews can contribute only one presence under the sentence-level top-1 framework. Even among multi-sentence reviews, 45.34% of visible-heritage records contained no T1/T2/T4 discourse.*

**Table S27. Heritage-related topic combinations in reviews with visible heritage imagery.**

| **Scope** | **Topic combination / academy** | **Reviews with expression** | **Eligible reviews** | **Conditional occurrence** | **95% CI** |
| --- | --- | --- | --- | --- | --- |
| Definition | T1/T4 union | 318 | 787 | 40.41% | 37.03%-43.87% |
| Definition | T1/T2/T4 union | 375 | 787 | 47.65% | 44.18%-51.14% |
| Definition | T1/T2/T3/T4 union | 629 | 787 | 79.92% | 76.98%-82.57% |
| Site | Yuelu | 246 | 446 | 55.16% | 50.52%-59.71% |
| Site | Bailudong | 60 | 159 | 37.74% | 30.57%-45.48% |
| Site | Songyang | 66 | 165 | 40.00% | 32.83%-47.62% |
| Site | Yingtian exploratory | 3 | 17 | 17.65% | 6.19%-41.03% |
| Sensitivity | Reiteration-screened main indicator | 366 | 787 | 46.51% | 43.05%–50.00% |

*Note. Percentages use the 787 reviews containing at least one Building space, Cultural symbol, or Environment landscape image as the denominator. The T1/T2/T4 union occurs in 375 reviews (47.65%); the broader T1/T2/T3/T4 union occurs in 629 (79.92%). Table S26 presents sentence-count groups. Yingtian contributes 17 eligible reviews.*

**Table S28. Architectural/spatial and heritage-related discourse overlap.**

| **T3 architectural/spatial discourse** | **T1/T2/T4 heritage-related discourse** | **Reviews** | **Share of 787** |
| --- | --- | --- | --- |
| Present | Absent | 254 | 32.27% |
| Absent | Present | 257 | 32.66% |
| Present | Present | 118 | 14.99% |
| Absent | Absent | 158 | 20.08% |
| Total | — | 787 | 100.00% |

*Note. Of 787 visible-heritage reviews, 372 contain T3 and 375 contain T1/T2/T4. Both occur in 118, T3 alone in 254, and T1/T2/T4 alone in 257; the union contains 629 (79.92%).*

**Table S29. Logistic specifications for heritage-related discourse in core reviews with visible heritage imagery.**

| **Model** | **N** | **k** | **Negative LL** | **AIC** | **Specification** |
| --- | --- | --- | --- | --- | --- |
| M1 | 770 | 3 | 523.1924 | 1052.3848 | Site only |
| M2 | 770 | 5 | 520.1265 | 1050.2530 | Add length and image count |
| M3 | 770 | 10 | 518.3761 | 1056.7523 | Add dominant/tied visual category |

*Note. All three specifications model the T1/T2/T4 presence indicator in 770 core-site reviews with visible heritage imagery. The specification with site, review length, and image count has the lowest AIC among those compared. Adding dominant/tied visual categories provides a descriptive specification comparison, not evidence that visual content has no relationship with expression.*

**Table S30. Logistic model with academy, review length, and image count**

| **Predictor** | **OR** | **95% CI** | **p** |
| --- | --- | --- | --- |
| Songyang vs Bailudong | 1.067 | 0.681-1.672 | 0.778 |
| Yuelu vs Bailudong | 1.944 | 1.338-2.825 | <0.001 |
| Log review length | 1.101 | 0.931-1.302 | 0.259 |
| Log image count | 0.719 | 0.552-0.937 | 0.015 |

**Table S31. Logistic model additionally including dominant/tied visual category**

| **Predictor** | **OR** | **95% CI** | **p** |
| --- | --- | --- | --- |
| Songyang vs Bailudong | 1.077 | 0.680-1.705 | 0.752 |
| Yuelu vs Bailudong | 1.982 | 1.362-2.884 | <0.001 |
| Log review length | 1.118 | 0.944-1.325 | 0.196 |
| Log image count | 0.731 | 0.560-0.955 | 0.022 |
| Cultural symbol vs Building space | 1.223 | 0.849-1.760 | 0.280 |
| Environment landscape vs Building space | 1.148 | 0.657-2.005 | 0.628 |
| People activity vs Building space | 0.964 | 0.426-2.185 | 0.931 |
| Service access vs Building space | 0.404 | 0.078-2.082 | 0.278 |
| Tie vs Building space | 1.241 | 0.833-1.848 | 0.288 |

*Note. Adding dominant/tied visual categories increases AIC from 1050.253 to 1056.752. None of the displayed category coefficients is statistically distinguishable from the building-space reference at p<0.05 in this specification.*

**Table S32. Sensitivity of the heritage-related discourse–textual valence association.**

| **Specification** | **N** | **B** | **95% CI** | **p** | **R²** |
| --- | --- | --- | --- | --- | --- |
| Main indicator, all core matched reviews | 812 | 0.121 | 0.043-0.199 | 0.002 | 0.205 |
| Main indicator, core visible-cue reviews | 770 | 0.112 | 0.032-0.192 | 0.006 | 0.186 |
| T1/T4 union | 812 | 0.110 | 0.031-0.188 | 0.006 | 0.203 |
| T1/T2/T3/T4 union | 812 | 0.142 | 0.025-0.260 | 0.018 | 0.202 |
| Main indicator; probability-weighted valence outcome | 812 | 0.116 | 0.042-0.190 | 0.002 | 0.217 |
| Main indicator; positive-sentence share outcome | 812 | 0.098 | 0.044-0.152 | <0.001 | 0.182 |
| Reiteration-screened main indicator | 812 | 0.129 | 0.051–0.207 | 0.001 | 0.206 |

*Note. Each row reports the focal coefficient from an OLS model adjusted for T6 service- and access-related discourse, log review length, log image count, academy, dominant/tied visual category, and period, with HC3 robust standard errors. Unless otherwise stated, the outcome is complete-review textual valence. Alternative topic unions change observed content coverage, not the degree of a psychological construct. The repetition-screened indicator excludes the 19 sentences flagged in Table S40. All coefficients are observational associations.*

**Table S33. OLS estimates for complete-review textual valence.**

| **Predictor** | **B** | **HC3 SE** | **95% CI** | **p** |
| --- | --- | --- | --- | --- |
| Intercept | 0.843 | 0.129 | [0.590, 1.096] | <0.001 |
| Heritage-related discourse (T1/T2/T4 presence) | 0.121 | 0.040 | [0.043, 0.199] | 0.002 |
| T6 service- and access-related discourse | -0.490 | 0.058 | [-0.603, -0.377] | <0.001 |
| Log review length | -0.086 | 0.022 | [-0.129, -0.043] | <0.001 |
| Log image count | 0.011 | 0.037 | [-0.061, 0.082] | 0.769 |
| Songyang vs Bailudong | -0.013 | 0.065 | [-0.140, 0.113] | 0.836 |
| Yuelu vs Bailudong | 0.075 | 0.051 | [-0.026, 0.175] | 0.144 |
| Cultural symbol vs Building space | -0.041 | 0.048 | [-0.134, 0.053] | 0.392 |
| Environment landscape vs Building space | -0.008 | 0.078 | [-0.161, 0.144] | 0.917 |
| People/activity context vs Building space | -0.104 | 0.080 | [-0.261, 0.053] | 0.196 |
| Service/access cue vs Building space | -0.008 | 0.169 | [-0.338, 0.323] | 0.964 |
| Tie vs Building space | 0.008 | 0.051 | [-0.092, 0.107] | 0.876 |
| Period 2020-2022 vs 2016-2019 | 0.046 | 0.065 | [-0.082, 0.173] | 0.482 |
| Period 2023-2026 vs 2016-2019 | 0.107 | 0.058 | [-0.007, 0.221] | 0.066 |

*Note. Textual valence=(positive sentences−negative sentences)/valid sentences. Estimates use HC3 robust standard errors; N=812, R²=0.204786, adjusted R²=0.191832. Reference categories are Bailudong, Building space, and 2016–2019.*

**Table S34. Component-specific associations with complete-review textual valence.**

| **Model** | **Component term** | **N** | **B** | **HC3 SE** | **95% CI** | **p** |
| --- | --- | --- | --- | --- | --- | --- |
| T1 only | T1 Historical-cultural discourse | 812 | 0.058 | 0.045 | [-0.031, 0.147] | 0.199 |
| T2 only | T2 Discourse on scholarly atmosphere | 812 | 0.118 | 0.049 | [0.022, 0.213] | 0.016 |
| T4 only | T4 Interpretation- and knowledge-related discourse | 812 | 0.097 | 0.046 | [0.006, 0.188] | 0.036 |
| Joint components | T1 Historical-cultural discourse | 812 | 0.071 | 0.046 | [-0.019, 0.161] | 0.124 |
| Joint components | T2 Discourse on scholarly atmosphere | 812 | 0.123 | 0.048 | [0.028, 0.218] | 0.011 |
| Joint components | T4 Interpretation- and knowledge-related discourse | 812 | 0.110 | 0.047 | [0.017, 0.203] | 0.020 |

*Note. In the core matched sample, T1 occurs in 185 reviews (22.78%), T2 in 90 (11.08%), T4 in 178 (21.92%), and at least one component in 400 (49.26%). Single-component models enter only the named T1/T2/T4 predictor, without defining mutually exclusive groups. The joint model enters all three predictors. Models adjust for T6, log review length, log image count, academy, dominant/tied visual category, and period, with HC3 robust standard errors. The joint Wald test is χ²(3)=12.45, p=0.006; maximum VIF=1.93. Pairwise coefficient contrasts are nonsignificant (all p≥0.440), so the estimates do not establish a stronger association for any one component.*

**Table S35. Heritage-related discourse and service/access interaction models.**

**Panel A. T1/T2/T4 presence by T6 presence**

| **T1/T2/T4 presence** | **T6** | **Full core matched N** | **Remaining-text eligible core matched N** |
| --- | --- | --- | --- |
| 0 | 0 | 308 | 308 |
| 1 | 0 | 327 | 132 |
| 0 | 1 | 104 | 104 |
| 1 | 1 | 73 | 73 |
| Total |  | 812 | 617 |

**Panel B. Interaction models**

| **Model/outcome** | **Term** | **N** | **B** | **HC3 SE** | **95% CI** | **p** |
| --- | --- | --- | --- | --- | --- | --- |
| Full core matched, full-review text valence | Heritage discourse | 812 | 0.0850 | 0.0415 | 0.0037 to 0.1662 | 0.0405 |
| Full core matched, full-review text valence | T6 | 812 | −0.5612 | 0.0829 | −0.7236 to −0.3987 | <0.001 |
| Full core matched, full-review text valence | T1/T2/T4 presence × T6 | 812 | 0.1693 | 0.1073 | −0.0410 to 0.3796 | 0.1146 |
| Remaining-text eligible, remaining-text valence | Heritage discourse | 617 | 0.0362 | 0.0518 | −0.0653 to 0.1377 | 0.4844 |
| Remaining-text eligible, remaining-text valence | T6 | 617 | −0.5598 | 0.0835 | −0.7235 to −0.3961 | <0.001 |
| Remaining-text eligible, remaining-text valence | T1/T2/T4 presence × T6 | 617 | 0.0216 | 0.1249 | −0.2231 to 0.2664 | 0.8625 |

*Note. Models retain the image-adjusted covariates and reference categories. Each main coefficient is conditional on the other binary predictor equaling zero. The interaction tests whether the discourse–valence association varies by T6 presence; p=0.115 for complete text and p=0.862 for remaining text.*

**Table S36. Full-review and remaining-text valence on the same eligible sample**

**Panel A. Sample restriction and outcome reconstruction**

| **Model/outcome** | **Term** | **N** | **B** | **HC3 SE** | **95% CI** | **p** |
| --- | --- | --- | --- | --- | --- | --- |
| Core matched; complete text | Heritage discourse | 812 | 0.1212 | 0.0396 | 0.0435 to 0.1988 | 0.0022 |
| Core matched; complete text | T6 | 812 | −0.4900 | 0.0577 | −0.6032 to −0.3769 | <0.001 |
| Eligible sample; complete text | Heritage discourse | 617 | 0.1414 | 0.0448 | 0.0537 to 0.2291 | 0.0016 |
| Eligible sample; complete text | T6 | 617 | −0.4859 | 0.0592 | −0.6020 to −0.3699 | <0.001 |
| R3: same eligible sample, remaining-text valence | Heritage discourse | 617 | 0.0432 | 0.0524 | −0.0596 to 0.1459 | 0.4105 |
| R3: same eligible sample, remaining-text valence | T6 | 617 | −0.5516 | 0.0637 | −0.6764 to −0.4268 | <0.001 |
| Paired coefficient difference | Complete-review minus remaining-text T1/T2/T4 coefficient | 617 | 0.0982 | 0.0250 | 0.049 to 0.147 | <0.001 |

**Panel B. Alternative remaining-text outcomes**

| **Model/outcome** | **Term** | **N** | **B** | **HC3 SE** | **95% CI** | **p** |
| --- | --- | --- | --- | --- | --- | --- |
| Remaining-text probability valence | Heritage discourse | 617 | 0.0341 | 0.0499 | −0.0637 to 0.1319 | 0.4949 |
| Remaining-text probability valence | T6 | 617 | −0.5496 | 0.0614 | −0.6698 to −0.4293 | <0.001 |
| Remaining-text positive-sentence share | Heritage discourse | 617 | 0.0219 | 0.0384 | −0.0533 to 0.0971 | 0.5685 |
| Remaining-text positive-sentence share | T6 | 617 | −0.2496 | 0.0380 | −0.3242 to −0.1751 | <0.001 |

*Note. All models use T6, log review length, log image count, academy, dominant/tied visual category, and period, with HC3 robust standard errors. The two N=617 outcome models use identical reviews and predictors. Their formal within-review coefficient difference is ΔB=0.0982 (SE=0.0250, 95% CI 0.049–0.147, p<0.001).*

**Table S37. Platform rating models and proportional-odds diagnostics**

**Panel A. Focal heritage-related discourse coefficients for original platform ratings**

| **Model** | **N** | **B** | **Robust SE** | **95% CI(B)** | **OR** | **p** |
| --- | --- | --- | --- | --- | --- | --- |
| R1 pooled ordered (descriptive only) | 2,798 | 0.039 | 0.100 | [-0.156, 0.235] | 1.040 | 0.694 |
| R1 OLS | 2,798 | 0.024 | 0.031 | [-0.037, 0.085] | — | 0.436 |
| R2 pooled ordered (secondary only) | 812 | 0.211 | 0.179 | [-0.140, 0.562] | 1.235 | 0.239 |
| R2 OLS | 812 | 0.055 | 0.051 | [-0.045, 0.154] | — | 0.281 |

*Note. The core text-sample specification adjusts for T6, log(1+sentence count), academy, and period. The core matched specification additionally uses the matched-sample covariates defined in the main text. Pooled ordered-logit estimates are retained only as descriptive diagnostics because the global proportional-odds assumption is rejected in the core text sample. The four native cumulative thresholds in Panel C constitute the principal rating inference; OLS on the 1–5 scale is retained as a sensitivity analysis.*

**Panel B. Parallel-lines diagnostic outcomes**

| **Scope** | **Equality test** | **Wald χ²** | **df** | **p** | **Status** |
| --- | --- | --- | --- | --- | --- |
| R1 | All slopes | 145.902 | 21 | <0.001 | Precision warning retained |
| R1 | T1/T2/T4 presence slopes | 6.845 | 3 | 0.077 | Precision warning retained |
| R2 | All slopes | Not interpretable | 39 | Not interpretable | Failed/unstable |
| R2 | T1/T2/T4 presence slopes | Not interpretable | 3 | Not interpretable | Failed/unstable |

**Panel C. Heritage-related discourse coefficients at each original rating threshold in the core text sample**

| **Threshold** | **Lower / higher n** | **B** | **Cluster SE** | **95% CI(B)** | **p** |
| --- | --- | --- | --- | --- | --- |
| rating>1 | 69 / 2,729 | 0.516 | 0.329 | [-0.130, 1.161] | 0.117 |
| rating>2 | 91 / 2,707 | 0.040 | 0.259 | [-0.468, 0.548] | 0.877 |
| rating>3 | 245 / 2,553 | 0.104 | 0.172 | [-0.233, 0.441] | 0.544 |
| rating>4 | 756 / 2,042 | -0.004 | 0.101 | [-0.202, 0.194] | 0.971 |

*Note. The stacked cumulative-logit diagnostic clusters each review's four threshold rows. In the core text sample, the global parallel-lines test rejects the proportional-odds assumption (chi-square(21)=145.902, p<0.001), while the T1/T2/T4-specific slope-equality test is not significant (chi-square(3)=6.845, p=0.077). Across the four native cut points, the heritage-related-discourse coefficients are B=0.516 (p=0.117), 0.040 (p=0.877), 0.104 (p=0.544), and −0.004 (p=0.971); every 95% CI includes zero. The HC3 OLS sensitivity estimate is B=0.024 (95% CI −0.037–0.085, p=0.436). Sparse lower ratings make the 812-review matched rating diagnostic secondary and unstable. No rating categories were collapsed.*

**Table S38. Associations between all seven topics and platform ratings**

**Panel A. Predefined omnibus tests**

| **Model** | **Test** | **Wald χ²** | **df** | **p** |
| --- | --- | --- | --- | --- |
| ordered | All seven = 0 | 245.306 | 7 | <0.001 |
| ordered | All seven equal | 244.772 | 6 | <0.001 |
| OLS | All seven = 0 | 224.048 | 7 | <0.001 |
| OLS | All seven equal | 222.205 | 6 | <0.001 |

**Panel B. Complete ordered-model topic family; N=2,798**

| **Topic** | **Present n (%)** | **B** | **95% CI(B)** | **OR** | **p** | **BH q** |
| --- | --- | --- | --- | --- | --- | --- |
| T1 | 383 (13.7%) | -0.090 | [-0.377, 0.197] | 0.914 | 0.539 | 0.539 |
| T2 | 288 (10.3%) | 0.865 | [0.511, 1.219] | 2.375 | <0.001 | <0.001 |
| T3 | 827 (29.6%) | 0.772 | [0.464, 1.080] | 2.165 | <0.001 | <0.001 |
| T4 | 543 (19.4%) | 0.930 | [0.585, 1.274] | 2.534 | <0.001 | <0.001 |
| T5 | 578 (20.7%) | 0.831 | [0.539, 1.124] | 2.296 | <0.001 | <0.001 |
| T6 | 435 (15.5%) | -1.219 | [-1.499, -0.940] | 0.295 | <0.001 | <0.001 |
| T7 | 544 (19.4%) | 0.361 | [0.063, 0.659] | 1.435 | 0.018 | 0.021 |

**Panel C. Complete OLS sensitivity topic family**

| **Topic** | **B** | **HC3 SE** | **95% CI(B)** | **p** | **BH q** |
| --- | --- | --- | --- | --- | --- |
| T1 | 0.079 | 0.058 | [-0.036, 0.193] | 0.178 | 0.178 |
| T2 | 0.386 | 0.055 | [0.278, 0.495] | <0.001 | <0.001 |
| T3 | 0.397 | 0.060 | [0.280, 0.514] | <0.001 | <0.001 |
| T4 | 0.361 | 0.063 | [0.237, 0.484] | <0.001 | <0.001 |
| T5 | 0.391 | 0.053 | [0.288, 0.494] | <0.001 | <0.001 |
| T6 | -0.582 | 0.071 | [-0.721, -0.444] | <0.001 | <0.001 |
| T7 | 0.160 | 0.060 | [0.044, 0.277] | 0.007 | 0.008 |

*Note. All seven topic indicators enter simultaneously, with log(1+sentence count), academy, and period. Because the global proportional-odds assumption fails for the core rating model, the pooled ordered coefficients in Panel B are exploratory only and are not used to rank topic-specific associations. The OLS topic family in Panel C is retained as a sensitivity analysis. These topic-family results do not alter the principal threshold-specific finding that T1/T2/T4 heritage-related discourse is not stably associated with higher platform ratings.*

**Table S39. Site-associated heterogeneity in the heritage-related discourse–rating association**

**Panel A. Overall interaction test**

| **Test** | **N** | **Wald χ²** | **df** | **p** |
| --- | --- | --- | --- | --- |
| T1/T2/T4 presence × site coefficients jointly zero | 2,798 | 14.299 | 2 | <0.001 |

**Panel B. Conditional focal coefficients in the ordered rating model**

| **Site** | **B** | **HC1 SE** | **95% CI(B)** | **OR** | **95% CI(OR)** | **p** |
| --- | --- | --- | --- | --- | --- | --- |
| Bailudong | -0.441 | 0.211 | [-0.855, -0.027] | 0.643 | [0.425, 0.973] | 0.037 |
| Songyang | -0.407 | 0.224 | [-0.847, 0.032] | 0.665 | [0.429, 1.033] | 0.069 |
| Yuelu | 0.308 | 0.122 | [0.068, 0.547] | 1.360 | [1.070, 1.729] | 0.012 |

*Note. This site-interaction model is retained as a secondary diagnostic only. The pooled ordered-rating specification violates the global proportional-odds assumption (Table S37), so the interaction result is not used to construct the main theoretical account. Site-associated coefficient differences do not identify effects of unmeasured visitor characteristics, route design, interpretation, management, or environmental conditions.*

**Table S40. Repeated wording in heritage-related sentences and indicator sensitivity.**

**Panel A. Sentence-level screen**

| **Topic component** | **Sentences screened** | **Exact-repeat flags** | **Additional near-repeat flags** | **Total flagged** | **Flagged share** |
| --- | --- | --- | --- | --- | --- |
| T1 Historical-cultural discourse | 455 | 2 | 1 | 3 | 0.66% |
| T2 Discourse on scholarly atmosphere | 314 | 14 | 2 | 16 | 5.10% |
| T4 Interpretation- and knowledge-related discourse | 597 | 0 | 0 | 0 | 0.00% |
| Total | 1,366 | 16 | 3 | 19 | 1.39% |

**Panel B. Review-level indicator sensitivity**

| **Analytical sample** | **Reviews N** | **Original indicator-positive n (%)** | **Screened indicator-positive n (%)** | **Change (percentage points)** |
| --- | --- | --- | --- | --- |
| All matched reviews | 829 | 403 (48.61%) | 394 (47.53%) | −1.09 |
| Visible-heritage matched reviews | 787 | 375 (47.65%) | 366 (46.51%) | −1.14 |
| Core matched reviews | 812 | 400 (49.26%) | 391 (48.15%) | −1.11 |
| Core visible-cue reviews | 770 | 372 (48.31%) | 363 (47.14%) | −1.17 |

*Note. All 1,366 sentences assigned to T1, T2, or T4 were screened. Text was normalized using Unicode NFKC, converted to lowercase, and stripped of spaces and punctuation while retaining Chinese characters, Latin letters, and digits. Only sentences from different reviews within the same academy were compared. A flag required either an exact normalized match of at least 15 characters, or two normalized strings of at least 20 characters with a shorter-to-longer length ratio ≥0.75 and character four-gram Jaccard similarity ≥0.80. Nineteen sentences from 19 reviews formed six wording clusters: 16 exact matches and three additional near-matches. Removing flagged sentences and recomputing the indicator reclassified 18 of 1,141 indicator-positive reviews in the sentence-linked corpus, including nine reviews in each analytical subset in Panel B. No labels were recoded. The screen identifies highly repeated wording within the collected corpus; it cannot establish copying, an external source, factual accuracy, or internal understanding.*

**Table S41. Full core text and matched multimodal sample comparison.**

**Panel A. Sample characteristics**

| **Characteristic** | **Full core text, N=2,798** | **Core matched, N=812** |
| --- | --- | --- |
| Bailudong | 458 (16.37%) | 163 (20.07%) |
| Songyang | 523 (18.69%) | 171 (21.06%) |
| Yuelu | 1,817 (64.94%) | 478 (58.87%) |
| Pre-2020 | 338 (12.08%) | 146 (17.98%) |
| 2020–2022 | 966 (34.52%) | 195 (24.01%) |
| 2023–2026 | 1,494 (53.40%) | 471 (58.00%) |
| Sentence count, mean (SD) | 1.576 (1.510) | 2.232 (2.284) |
| Review length in characters, mean (SD) | 41.674 (59.735) | 74.638 (90.345) |
| Full-review text valence, mean (SD) | 0.618 (0.630) | 0.577 (0.573) |
| T1/T2/T4 presence=1 | 1,122 (40.10%) | 400 (49.26%) |
| T6=1 | 435 (15.55%) | 177 (21.80%) |
| Image flag present | 813 (29.06%) | 812 (100.00%) |

**Panel B. Common simplified text-only comparison models**

| **Model** | **Term** | **N** | **B** | **HC3 SE** | **95% CI** | **p** |
| --- | --- | --- | --- | --- | --- | --- |
| All core text reviews | Heritage discourse | 2,798 | 0.0720 | 0.0225 | 0.0278 to 0.1161 | 0.0014 |
| All core text reviews | T6 | 2,798 | −0.7179 | 0.0437 | −0.8035 to −0.6323 | <0.001 |
| Core matched reviews | Heritage discourse | 812 | 0.1466 | 0.0388 | 0.0705 to 0.2227 | 0.0002 |
| Core matched reviews | T6 | 812 | −0.4694 | 0.0605 | −0.5880 to −0.3508 | <0.001 |

*Note. Review length is the sum of sentence-character counts and reproduces the matched-sample measure. Both comparison models specify text valence as a function of T1/T2/T4 presence, T6, log(1 + sentence count), academy, and period, with HC3 robust standard errors and Bailudong and pre-2020 references. These are common-specification sample comparisons rather than replacements for the image-adjusted model. Positive focal coefficients in both samples indicate directional consistency under this specification, without establishing random selection or representativeness. The matched sample’s 100% image presence follows from eligibility.*

**Table S42. Within-review sentence order and next-sentence sentiment**

**Panel A. Eligibility and global within-review permutation test**

| **Quantity** | **Result** |
| --- | --- |
| Core multi-sentence reviews | 753 of 2,798 |
| Excluded single-sentence reviews | 2,045 |
| Adjacent sentence pairs | 1,611 |
| Within-review permutations / seed | 5,000 / 20260902 |
| Global statistic / permutation p | 527.558 / 0.0019996 |

**Panel B. All 49 transitions: observed count / permutation-mean count**

| **From → To** | **T1** | **T2** | **T3** | **T4** | **T5** | **T6** | **T7** |
| --- | --- | --- | --- | --- | --- | --- | --- |
| T1 | 31 / 32.2 | 10 / 15.4 | 41 / 54.5 | 18 / 13.8 | 12 / 13.9 | 27 / 23.5 | 29 / 24.6 |
| T2 | 18 / 15.5 | 4 / 6.8 | 31 / 38.4 | 15 / 10.3 | 11 / 8.5 | 8 / 7.2 | 10 / 10.4 |
| T3 | 71 / 54.4 | 54 / 38.4 | 307 / 303.9 | 16 / 13.0 | 43 / 29.8 | 44 / 42.2 | 60 / 56.0 |
| T4 | 10 / 13.8 | 7 / 10.3 | 11 / 13.0 | 35 / 37.0 | 12 / 11.9 | 27 / 24.0 | 41 / 32.1 |
| T5 | 16 / 13.9 | 9 / 8.6 | 15 / 29.7 | 10 / 11.9 | 20 / 19.2 | 14 / 15.4 | 27 / 25.7 |
| T6 | 21 / 23.4 | 4 / 7.2 | 35 / 42.3 | 26 / 23.9 | 19 / 15.4 | 94 / 91.5 | 44 / 41.6 |
| T7 | 21 / 24.6 | 13 / 10.5 | 32 / 56.0 | 24 / 32.1 | 17 / 25.7 | 36 / 41.5 | 111 / 96.1 |

**Panel C. BH-FDR q values for the complete 49-cell family**

| **From → To** | **T1** | **T2** | **T3** | **T4** | **T5** | **T6** | **T7** |
| --- | --- | --- | --- | --- | --- | --- | --- |
| T1 | 0.842 | 0.335 | 0.069 | 0.437 | 0.762 | 0.667 | 0.621 |
| T2 | 0.691 | 0.467 | 0.326 | 0.237 | 0.691 | 0.867 | 1.000 |
| T3 | 0.010 | 0.003 | 0.762 | 0.667 | 0.010 | 0.842 | 0.742 |
| T4 | 0.535 | 0.534 | 0.736 | 0.762 | 1.000 | 0.691 | 0.185 |
| T5 | 0.742 | 1.000 | 0.003 | 0.762 | 0.856 | 0.842 | 0.856 |
| T6 | 0.762 | 0.437 | 0.428 | 0.762 | 0.586 | 0.762 | 0.791 |
| T7 | 0.667 | 0.691 | 0.003 | 0.204 | 0.105 | 0.586 | 0.010 |

**Panel D. Focal next-sentence sentiment coefficient**

| **Term** | **Pairs / reviews** | **B** | **Cluster SE** | **95% CI(B)** | **p** |
| --- | --- | --- | --- | --- | --- |
| Current T1/T2/T4 presence | 1,611 / 753 | -0.020 | 0.043 | [-0.105, 0.065] | 0.647 |

*Note. Sentence order is writing order. The null distribution randomly reorders sentences within each review while preserving its sentence set and boundaries. T3→T1, T3→T2, T3→T5, and T7→T7 exceed the null mean, whereas T5→T3 and T7→T3 fall below it (all q<0.05). The focal model controls the current sentence’s sentiment code, log sentence count, academy, and period, with review-clustered standard errors. A T1/T2/T4 sentence is not followed by demonstrably more positive adjacent sentiment under this model. These analyses do not recover the temporal sequence of psychological experience.*

**Table S43. Sample eligibility and original platform rating distributions**

**Panel A. Distinct sample scopes**

| **Scope** | **Reviews** | **Additional count** |
| --- | --- | --- |
| Valid corpus | 2,855 | 4,531 valid sentences cover 2,854 reviews |
| Core text; primary rating model R1 | 2,798 | Bailudong 458; Songyang 523; Yuelu 1,817 |
| All matched text–image reviews | 829 | 6,933 cleaned linked images |
| Core matched; secondary rating model R2 | 812 | 6,772 cleaned linked images |
| Image projection scope | 835 | 6,975 projected images; distinct image-side scope |
| Core multi-sentence sequence sample | 753 | 1,611 adjacent pairs; 2,045 single-sentence reviews excluded |

**Panel B. Uncollapsed 1–5 star ratings**

| **Rating** | **Core text n** | **Share** | **Core matched n** | **Share** |
| --- | --- | --- | --- | --- |
| 1 | 69 | 2.47% | 11 | 1.35% |
| 2 | 22 | 0.79% | 3 | 0.37% |
| 3 | 154 | 5.50% | 30 | 3.69% |
| 4 | 511 | 18.26% | 176 | 21.67% |
| 5 | 2,042 | 72.98% | 592 | 72.91% |

*Note. The original platform rating is constant within each review and takes integer values from 1 to 5, with no missing or illegal values in the sentence-linked sample. Five-star shares are 72.98% in the core text sample and 72.91% in the core matched sample. Ratings are structured evaluations supplied by the same users on the same platform, not an independent psychological measure. The scopes in Panel A are distinct source sets; the unresolved matching difference is described in Table S3.*

**Table S44. EEQE direction and complete-review textual valence.**

| **Environmental-quality direction** | **Positive full-text valence** | **Zero full-text valence** | **Negative full-text valence** | **Total** |
| --- | --- | --- | --- | --- |
| Positive | 349 | 22 | 4 | 375 |
| Negative | 18 | 4 | 31 | 53 |
| Mixed | 14 | 1 | 2 | 17 |
| Neutral description | 1 | 1 | 0 | 2 |
| No explicit quality expression | 159 | 59 | 34 | 252 |
| Total | 541 | 87 | 71 | 699 |

*Note. Counts concern 699 determinate cases in the 700-review EEQE sample. Complete-review textual valence is the sign of (positive sentences−negative sentences)/valid sentences. Of 53 negative EEQE records, 18 have positive textual valence. Of 375 positive EEQE records, 22 have zero and four have negative textual valence. EEQE presence includes positive, negative, mixed, and neutral-descriptive evaluations.*
